# Supplementary material for: Glycerol metabolism triggers trypanosome differentiation into transmissible forms in mammalian tissue-like conditions
Source: Nat Commun. 2026 Jul 17;17:8766. doi: 10.1038/s41467-026-75670-w (PMC13493832; doi:10.1038/s41467-026-75670-w)
Supplement: Supplementary file 1 [file 41467_2026_75670_MOESM1_ESM.pdf]

Glycerol metabolism triggers trypanosome differentiation into transmissible forms in mammalian tissue-like conditions

Mohammad El Kadri[1], Fabien Guegan[2], Parul Sharma[3,4], Estefania Calvo Alvarez[3], Erika Pineda[1], Pauline Morand[1], Jean Marc Tsagmo Ngoune[3], Justine Escard[3,4], Jean-William Dupuy[5,6], Aline Rimoldi[1], Nicolas Plazolles[1], Edern Cahoreau[7,8], Marc Biran[9], Michael P. Barrett[10,11], Luisa M. Figueiredo[2], Brice Rotureau[3,12], Frédéric Bringaud[1#]

[1] Univ. Bordeaux, CNRS, Microbiologie Fondamentale et Pathogénicité (MFP), UMR 5234, F-33000 Bordeaux, France
[2] Gulbenkian Institute for Molecular Medicine, 1649-028 Lisbon, Portugal
[3] Trypanosome Transmission Group, Trypanosome Cell Biology Unit, Institut Pasteur, Université Paris Cité, INSERM U1347, Paris, France
[4] Sorbonne Université, ED515 Complexité du Vivant, Paris, France
[5] Centre de Génomique Fonctionnelle, Plateforme Protéome, Université de Bordeaux, 146 rue Léo Saignat, 33076 Bordeaux, France
[6] Univ. Bordeaux, CNRS, INSERM, TBM-Core, US5, UAR 3427, OncoProt, F-33000 Bordeaux, France
[7] Toulouse Biotechnology Institute (TBI), Université de Toulouse, CNRS, INRA, INSA, Toulouse, France
[8] MetaToul–MetaboHUB, Toulouse, France
[9] Univ. Bordeaux, CNRS, Centre de Résonance Magnétique des Systèmes Biologiques (CRMSB), UMR 5536, F-33000 Bordeaux, France
[10] Wellcome Centre for Integrative Parasitology, Institute of Infection, Immunity and Inflammation, College of Medical, Veterinary and Life Sciences, University of Glasgow, Glasgow, United Kingdom
[11] Glasgow Polyomics, Wolfson Wohl Cancer Research Centre, Garscube Campus, College of Medical Veterinary and Life Sciences, University of Glasgow, Glasgow, United Kingdom
[12] Parasitology Unit, Institut Pasteur of Guinea, Conakry, Guinea

[#] Corresponding author: frederic.bringaud@u-bordeaux.fr

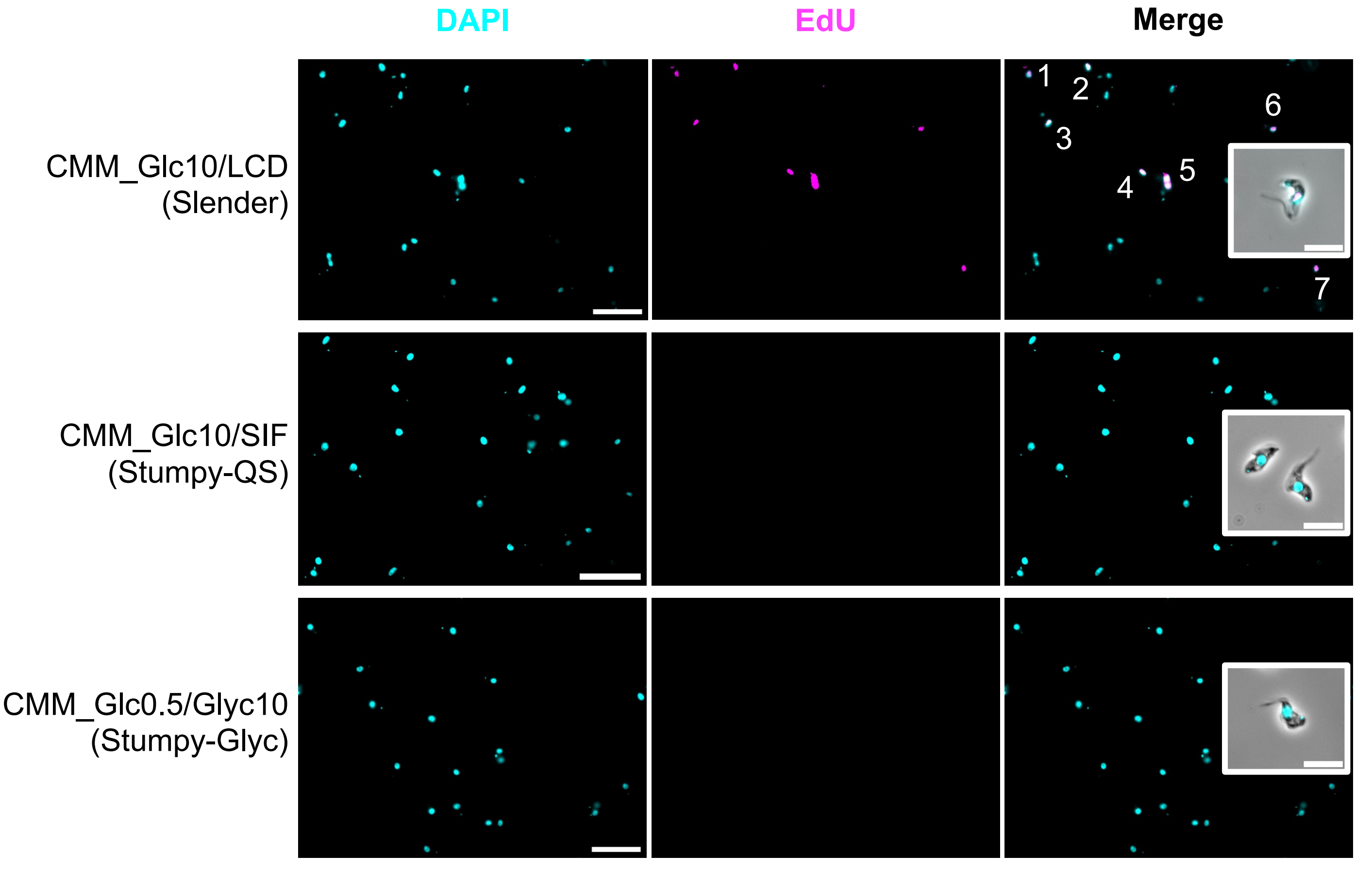

Supplementary figure 1. Stumpy-Glyc cells are cell cycle-arrested.
Microscopy images of the AnTat1.1E-GFP::PAD1$_{UTR}$ incubated in (*i*) CMM_Glc10 and maintained as slender forms by daily dilution to prevent high cell density (LCD), (*ii*) in SIF-rich CMM_Glc10 spent medium derived from a stationary phase culture of AnTat1.1E-GFP::PAD1$_{UTR}$ (CMM_Glc10/SIF) and (*iii*) in low glucose (0.5 mM) CMM containing 10 mM glycerol (CMM_Glc0.5/Glyc10), with EdU (false colored magenta) and DAPI (false colored cyan) (n = 3 independent experiments). Scale bar = 20 µm. Insets: representative images of labelled parasites (scale bar, 10 µm).

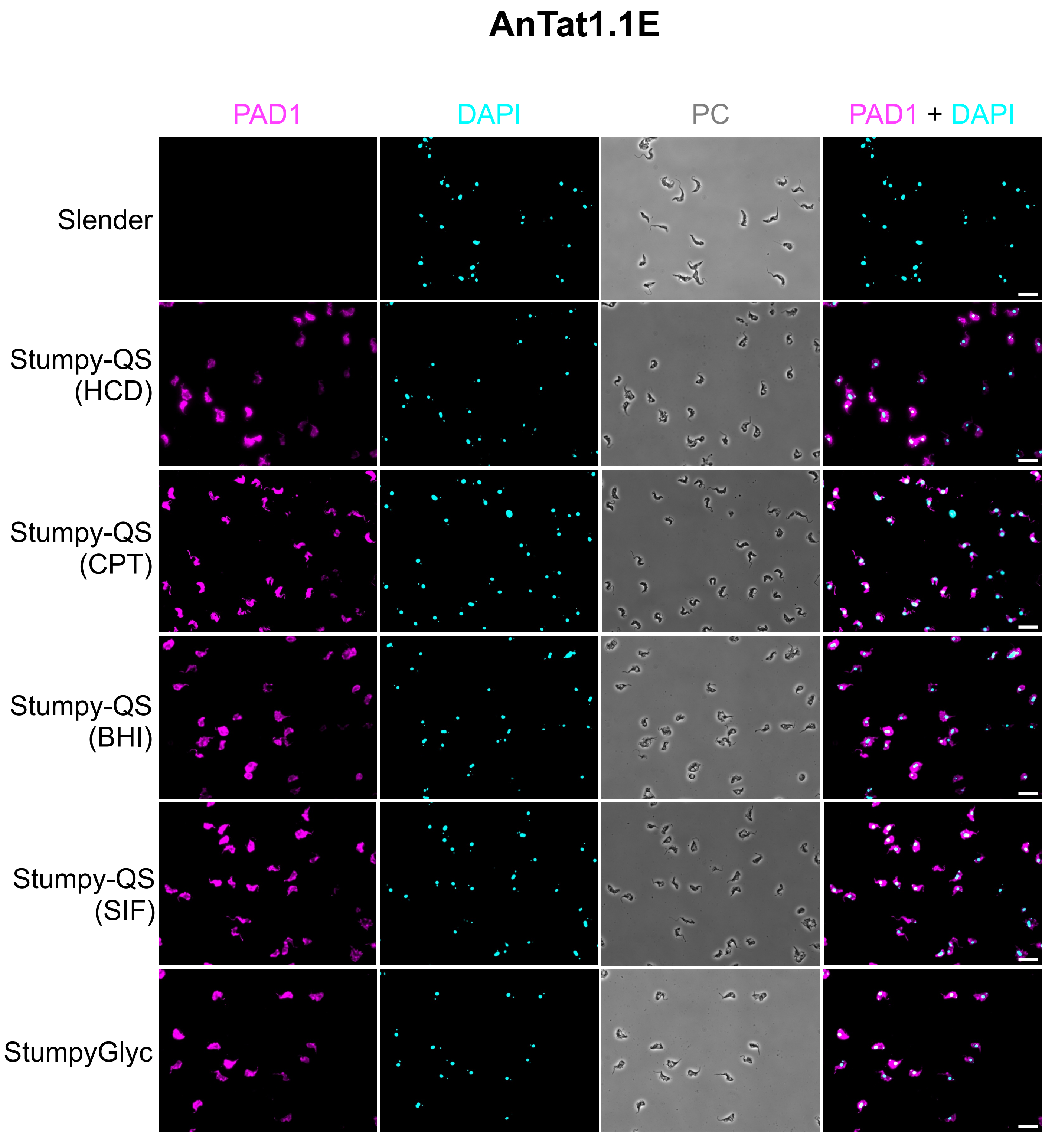

Supplementary figure 2. Stumpy-Glyc express PAD1 to the plasma membrane. Immunofluorescence analysis of the parental slender cells (AnTat1.1E) maintained at low cell density (*i*), stumpy-QS generated either at high cell density in CMM_Glc10 (HCD) (*ii*), in CMM_Glc10 containing 100 µM 8-pCPT-cAMP (CPT) (*iii*), in CMM_Glc10 containing 5% BHI medium (BHI) (*iv*) and in SIF-rich CMM_Glc10 spent medium derived from a stationary phase culture of AnTat1.1E-GFP::$PAD1_{UTR}$ (SIF) (*v*), as well as stumpy-Glyc generated in low glucose (0.5 mM) CMM containing 10 mM glycerol (*vi*). PAD1 expression is revealed using anti-PAD1 (false colored magenta), nucleus and kinetoplast using DAPI (false colored Cyan). PC Phase contrast. Scale bar = 20 µm.

Kinetoplast/Nucleus distance

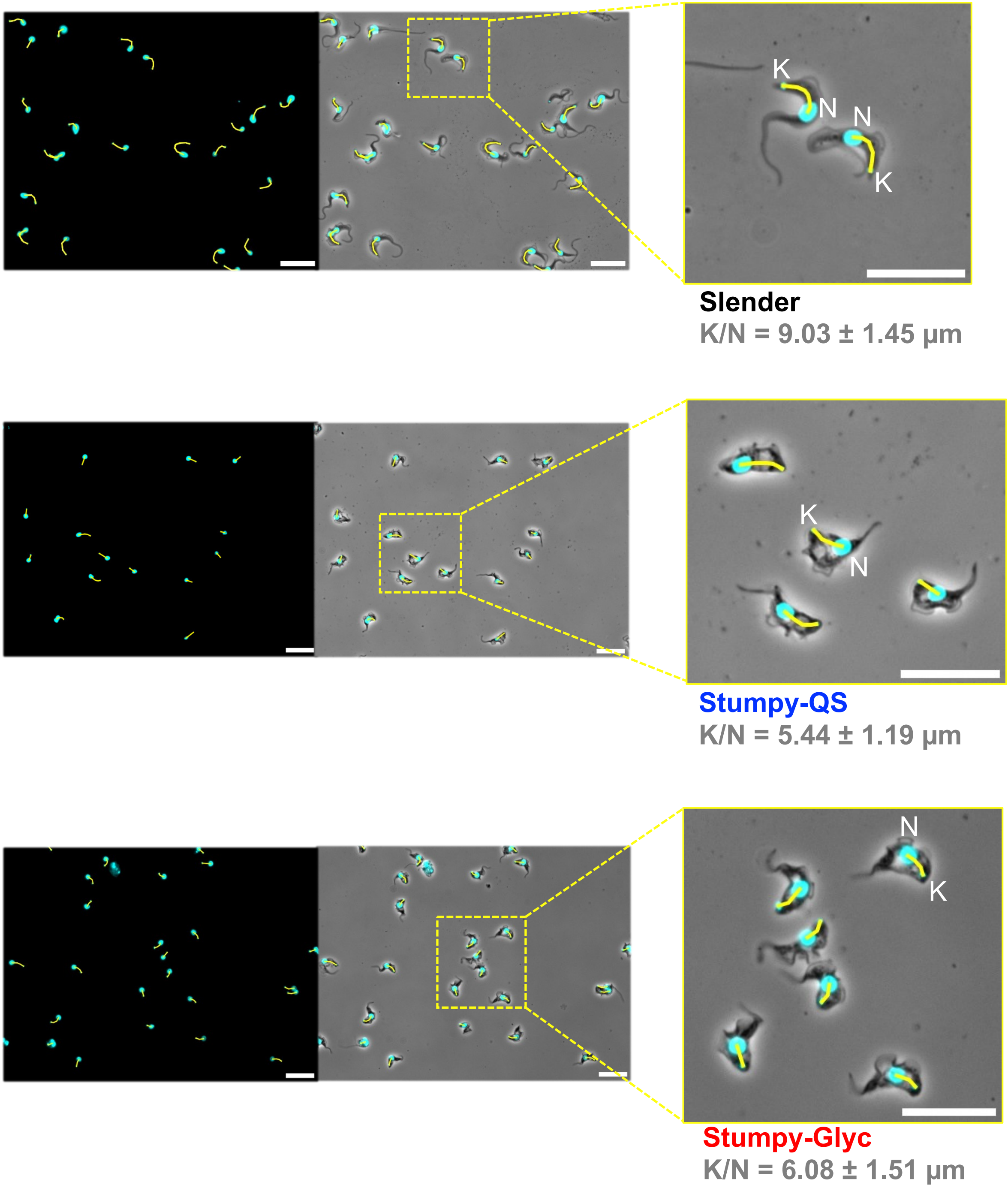

Supplementary figure 3. Stumpy-Glyc cells present a stumpy morphology.
Example PC Phase contrast images of slender, stumpy-QS and stumpy-Glyc stained with DAPI for nucleus (N) and kinetoplast (K) localization. Yellow lines indicate how the distance between the nucleus and kinetoplast was measured manually using ImageJ software. White scale bars = 20 µm.

	A	B	C	D	E	F	G
Upregulated proteins relative to slender forms (CMM_Glc10)	**131**	**144**	**109**	**110**	**73**	**139**	**100**
Downregulated proteins relative to slender forms (CMM_Glc10)	**108**	**79**	**59**	**80**	**64**	**99**	**53**

**A**	Slender-Glyc (CMM_Glc4/Glyc0.5)
**B**	Slender-Glyc (CMM_Glc4/Glyc2)
**C**	Stumpy-Glyc (CMM_Glc0.5/Glyc10)
**D**	Stumpy-QS (CMM_Glc10/SIF)
**E**	Stumpy-QS (CMM_Glc10/BHI)
**F**	Stumpy-QS (CMM_Glc10/HCD)
**G**	Procyclic (SDM79)

Supplementary figure 4. Number of differentially regulated proteins across *T. brucei* life cycle stages relative to slender forms (CMM_Glc10)
This table displays the numbers of differentially upregulated and downregulated proteins—across various *T. brucei* life cycle stages—relative to slender forms (CMM_Glc10). The conditions include "slender-Glyc" (CMM_Glc4/Glyc0.5 and CMM_Glc4/Glyc2), "Stumpy-Glyc" (CMM_Glc0.5/Glyc10), "Stumpy-QS" (CMM_Glc10/SIF, CMM_Glc10/BHI, CMM_Glc10/HCD), and "Procyclic" (SDM79). The names of the proteins regulated in each form, with their accession numbers, are provided in an Excel table.

Overlap of upregulated proteins—across various *T. brucei* lifecycle forms—relative to slender forms (CMM_Glc10)

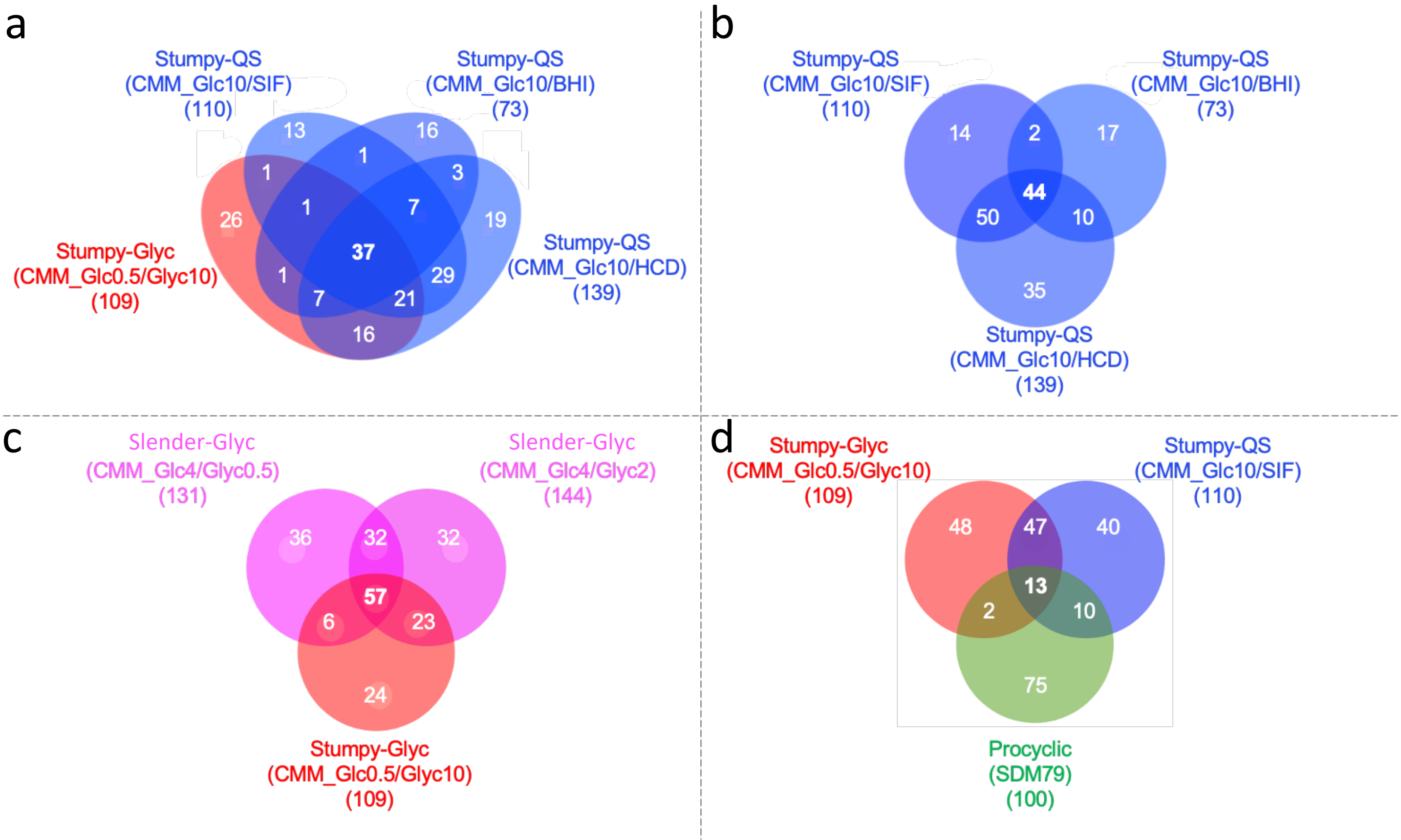

Overlap of downregulated proteins—across various *T. brucei* lifecycle forms—relative to slender forms (CMM_Glc10)

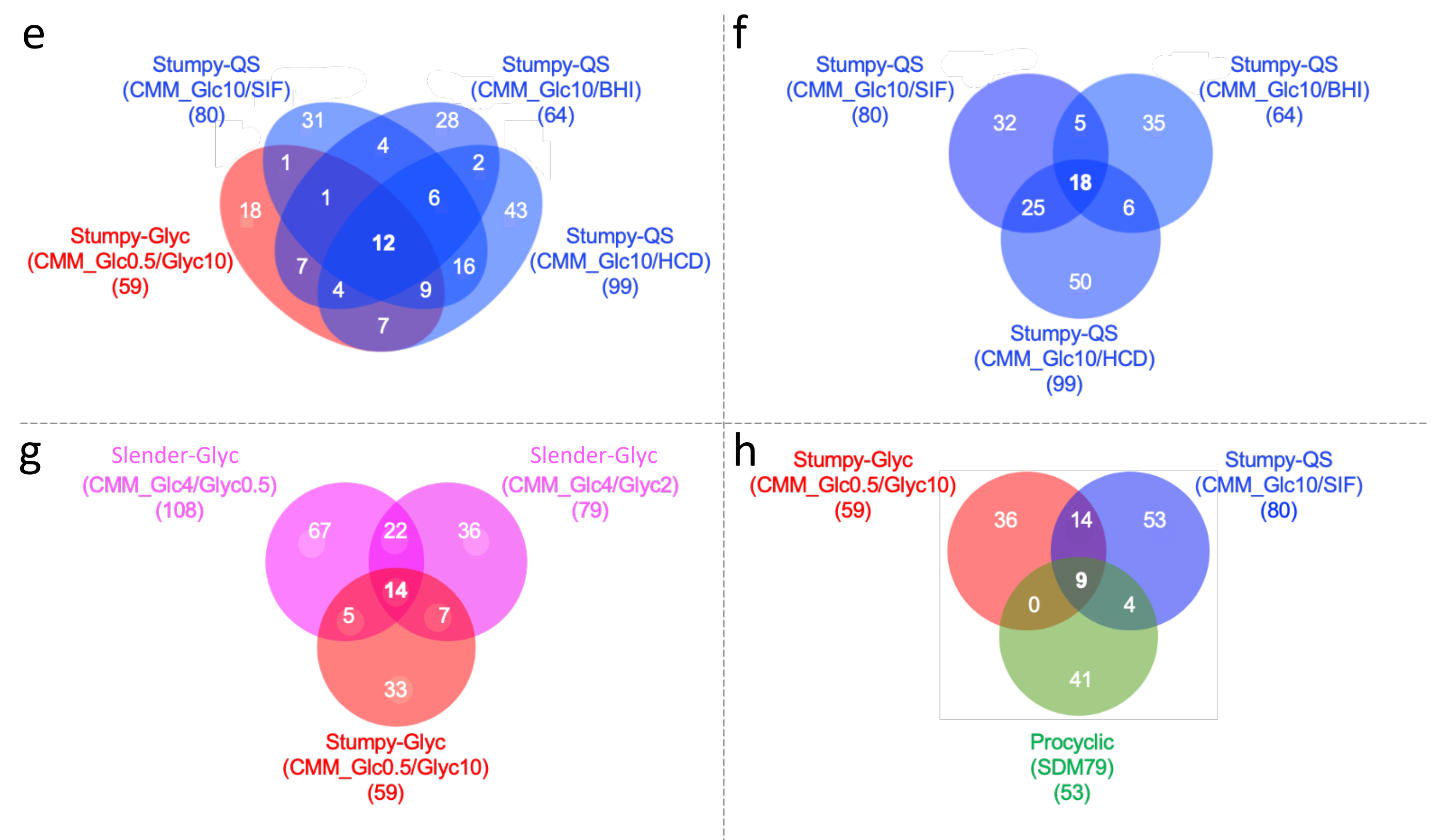

Supplementary figure 5. Stumpy-Glyc protein expression profile is closer to that of Stumpy-QS than to the PCF one.

This figure presents a series of Venn diagrams that illustrate the overlap of differentially regulated proteins, both upregulated (upper panel) and downregulated (lower panel)—across various *Trypanosoma brucei* life cycle stages—relative to the slender forms. For clarity, "regulated proteins" refers to proteins that are differentially regulated relative to the slender form (CMM_Glc10). The number of regulated proteins relative to slender forms is represented for each condition under the name in parentheses.

a, compares these upregulated proteins in the "Stumpy-Glyc" condition (CMM_Glc0.5/Glyc10) (red) with those in three "Stumpy-QS" conditions (blue): CMM_Glc10/SIF, CMM_Glc10/BHI, and CMM_Glc10/HCD. In this comparison, 37 proteins are consistently upregulated across all Stumpy-QS and Stumpy-Glyc conditions. **b,** shows the overlap of upregulated proteins among Stumpy-QS conditions alone, identifying 44 proteins commonly upregulated relative to slender forms. **c,** illustrates the overlap in upregulated proteins between the "Stumpy-Glyc" condition and two "slender-Glyc" conditions (pink), revealing 57 proteins upregulated across all these conditions. **d,** highlights the overlap of upregulated proteins across "Stumpy-Glyc" (red), SIF-induced "Stumpy-QS" (blue), and "Procyclic" (SDM79) (green) conditions, with only 13 proteins upregulated in all three. **e-h**, mirrors the upper panel but displays downregulated proteins instead of upregulated, relative to the slender forms.

The names of the proteins regulated in each form, with their accession numbers, are provided in the supplementary data 2.

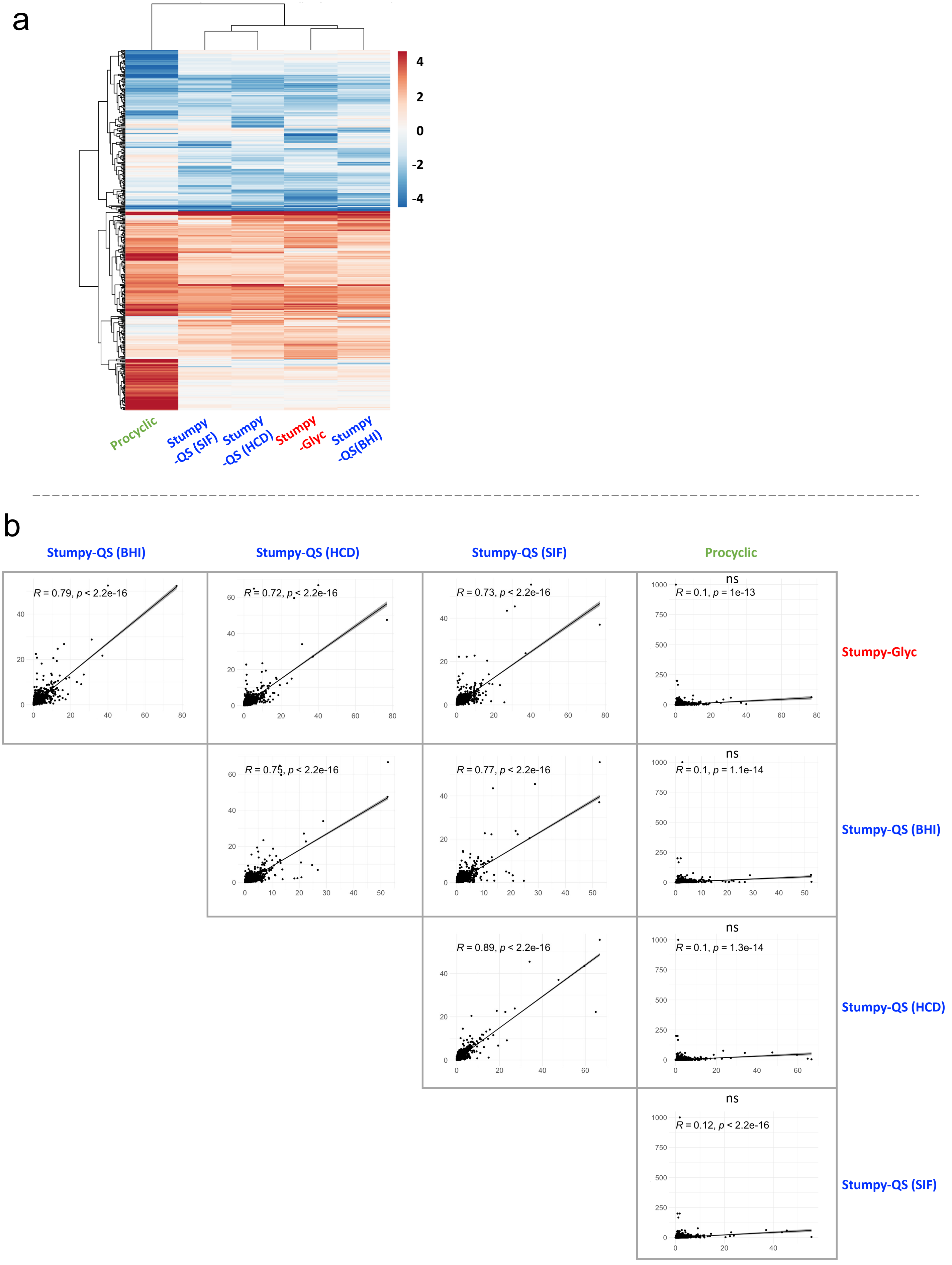
a
4
2
0
-2
-4
Procyclic
Stumpy -QS (SIF)
Stumpy -QS (HCD)
Stumpy -Glyc
Stumpy -QS(BHI)
b
Stumpy-QS (BHI)
Stumpy-QS (HCD)
Stumpy-QS (SIF)
Procyclic
Stumpy-Glyc
Stumpy-QS (BHI)
Stumpy-QS (HCD)
Stumpy-QS (SIF)
R = 0.79, p < 2.2e-16
R = 0.72, p < 2.2e-16
R = 0.73, p < 2.2e-16
ns
R = 0.1, p = 1e-13
R = 0.75, p < 2.2e-16
R = 0.77, p < 2.2e-16
ns
R = 0.1, p = 1.1e-14
R = 0.89, p < 2.2e-16
ns
R = 0.1, p = 1.3e-14
ns
R = 0.12, p < 2.2e-16

Supplementary figure 6. Comparison of the proteins significantly regulated under different induction conditions as compared to the slender forms.

a, A heat map encompassing all the significantly regulated proteins (greater than 2 logFC relative to slender form proteins), showing a comparative analysis of protein-level changes between conditions. **b**, Pearson correlation of protein fold-changes across different forms. Scatter plots show pairwise comparisons of log2-transformed fold changes of proteins between conditions. The solid line represents the linear regression fit with 95% confidence interval (shaded area). The Pearson correlation coefficient (r) and corresponding p-value are indicated on each plot. High positive r values indicate strong concordance in protein regulation between conditions, while low or negative r values indicate weak or inverse relationships. Pearson correlation coefficients (r) are shown on each plot; values ≥ 0.7 are considered strong correlations, whereas values below 0.7 are considered weak or not significant (ns).

Source data are provided as a Source Data file.

GO terms: fold enrichment (upregulated) compared to CMM_Glc10 (slenders)

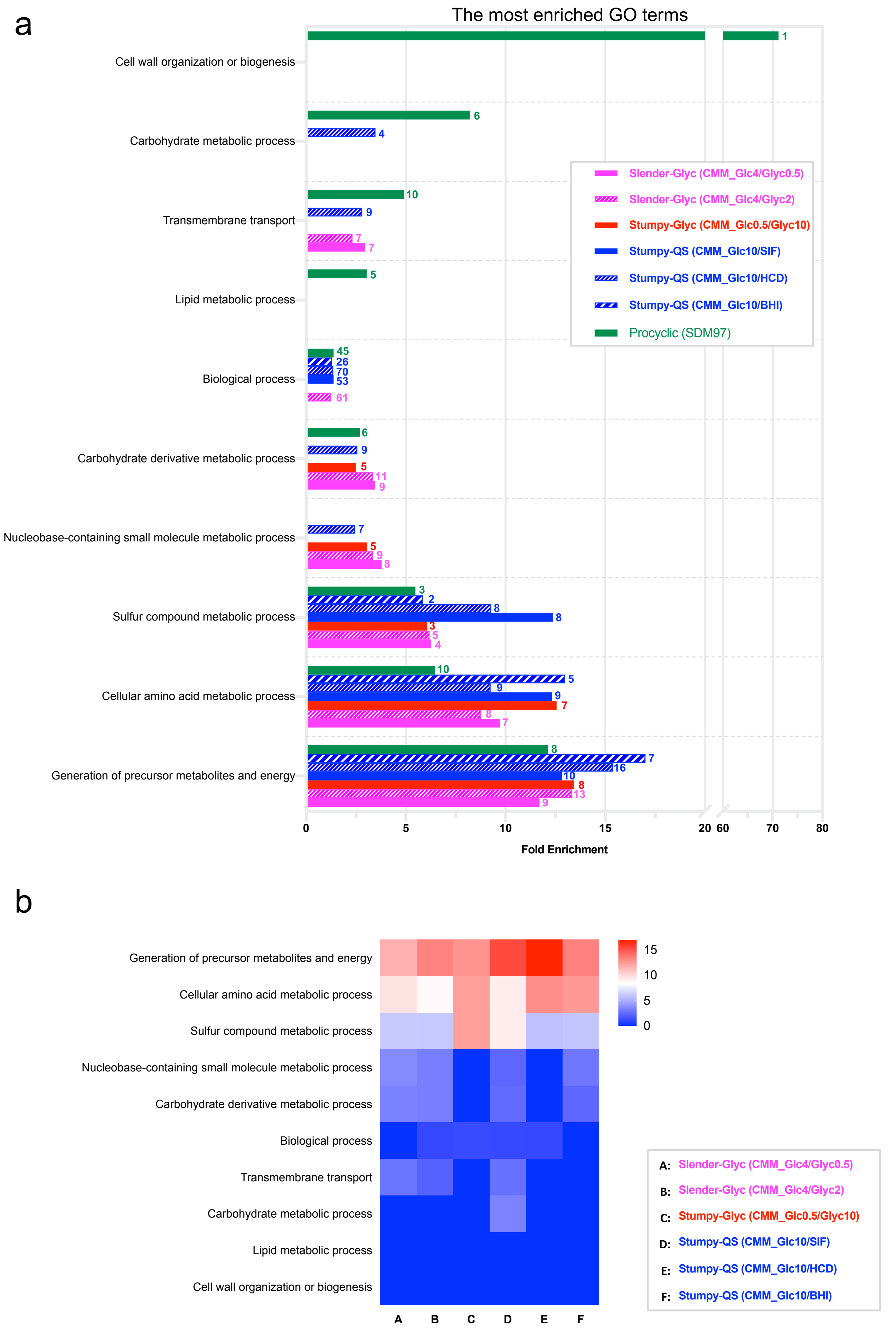

Supplementary figure 7. GO term enrichment analysis for biological processes of genes upregulated by >2 folds in different conditions as compared to slender forms (CMM_Glc10). **a**, shows terms on the y-axis that are significantly enriched (p<0.05) by more than two folds, comparing procyclic, stumpy-Glyc, stumpy-QS, and slender-Glyc forms. The numbers at the right end of the bars indicate the number of genes specific to each GO term. **b**, shows a heat map showing the enriched GO terms in different conditions compared to slender forms.
Source data are provided as a Source Data file.

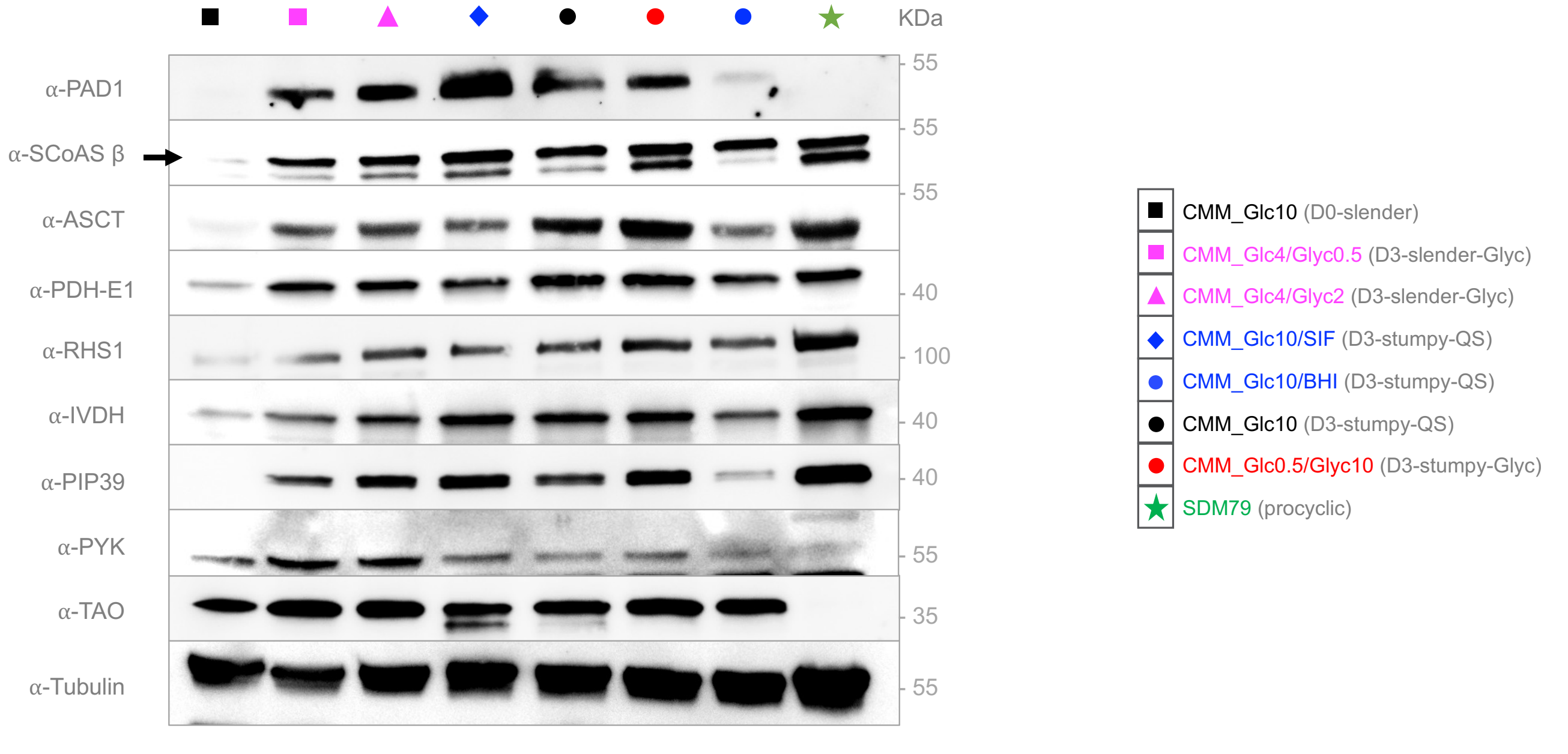

Supplementary figure 8. Stumpy-Glyc and slender-Glyc cells have a similar protein expression pattern as stumpy-QS.
Western blot analysis confirming the proteomics data using the different forms analyzed in the proteomics study, with a range of available antibodies.

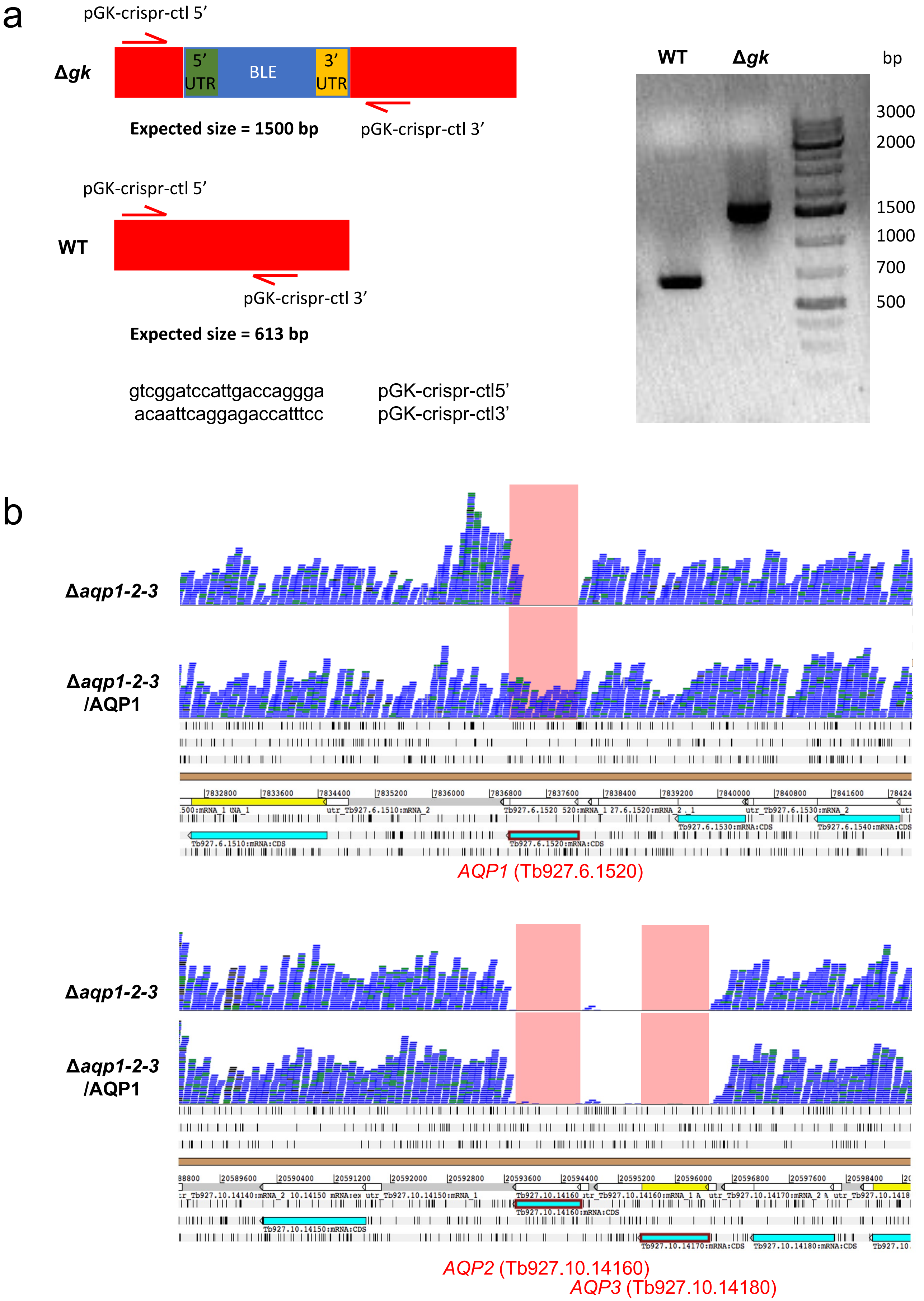
a
pGK-crispr-ctl 5’
Δgk
5’ UTR
BLE
3’ UTR
Expected size = 1500 bp
pGK-crispr-ctl 3’
pGK-crispr-ctl 5’
WT
pGK-crispr-ctl 3’
Expected size = 613 bp
gtcggatccattgaccaggga
acaattcaggagaccatttcc
pGK-crispr-ctl5’
pGK-crispr-ctl3’
WT
Δgk
bp
3000
2000
1500
1000
700
500
b
Δaqp1-2-3
Δaqp1-2-3 /AQP1
AQP1 (Tb927.6.1520)
Δaqp1-2-3
Δaqp1-2-3 /AQP1
AQP2 (Tb927.10.14160)
AQP3 (Tb927.10.14180)

Supplementary figure 9. Verification of successful gene inactivation/replacements of glycerol kinase (GK) and aquaglyceroporin (AQP) null mutants by PCR and genome sequencing.
a, Inactivation of the *GK* gene family. The left panel shows a schematic representation of one member of the 5 tandemly repeated *GK* genes, native (parental) or inactivated by insertion of the phleomycin resistance marker *Ble*R (Δ*gk*). The positions and sequences of the oligonucleotides (pGK-crispr-ctl5' and pGK-crispr-ctl3') used to verify gene inactivation by PCR are shown, along with the expected PCR product size. PCR products from the selected phleomycin-resistant and the parental cell lines are analyzed on an agarose gel (right panel). **b**, Replacement of the *AQP1-2-3* genes. Whole-genome sequencing data showing the absence of mapped reads across the *AQP1-2–3* genes in the aquaglyceroporin null mutant (Δ*aqp1-2-3*), while reads of the *AQP1* genes are present in the rescue cell line (Δ*aqp1-2-3*/AQP1). Sequencing reads were aligned to the parental AnTat1.1E reference genome.

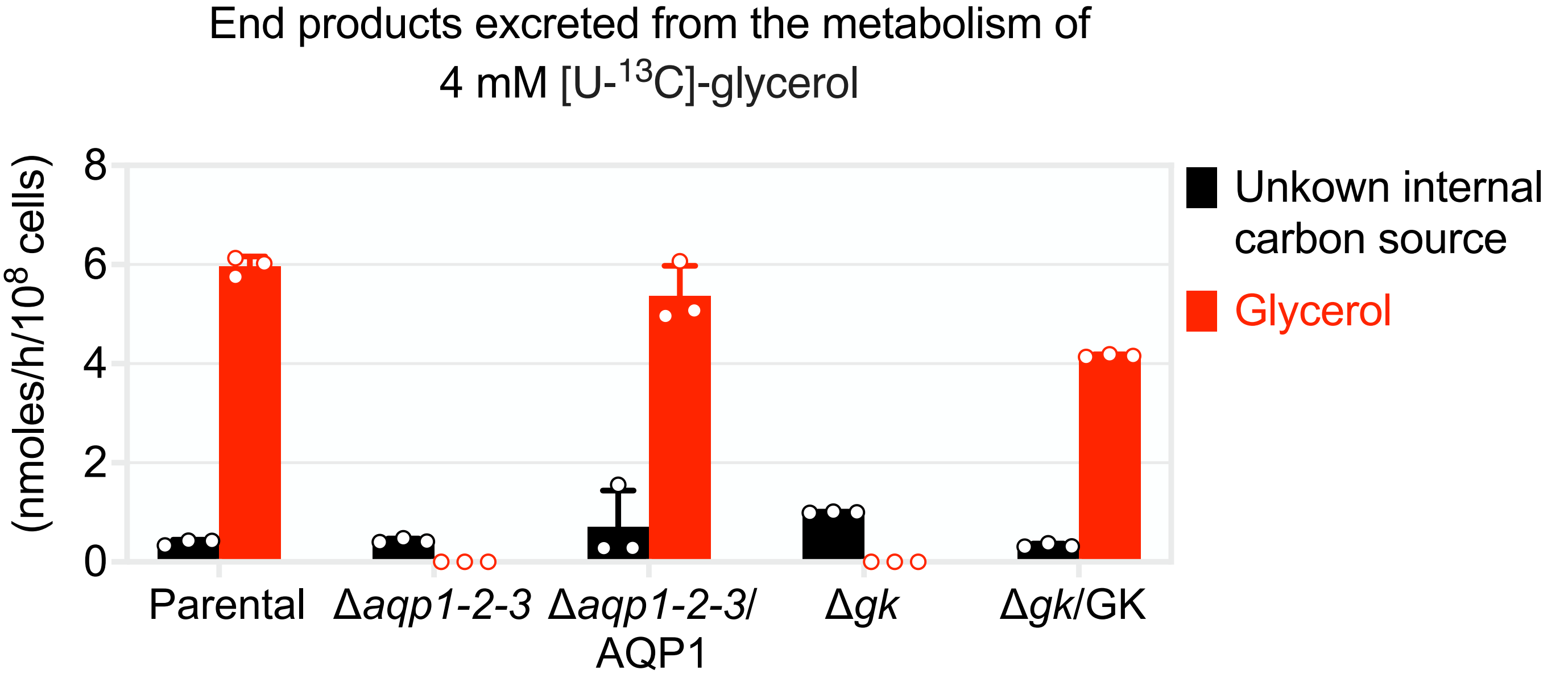

Supplementary figure 10. Validation of glycerol kinase and aquaglyceroporin null mutants and the corresponding rescue cell lines by ^{1}H-NMR analysis.
The parental, knockout (Δ*aqp1-2-3* and Δ*gk*) and rescue (Δ*aqp1-2-3*/AQP1 and Δ*gk*/GK) cell lines were incubated for 1 h in PBS/$NaHCO_3$ buffer containing 4 mM of uniformly ^{13}C-enriched glycerol ([U-^{13}C]-glycerol). The amounts of non-enriched and ^{13}C-enriched metabolic end products excreted in the spent medium from the metabolism of an unknown internal carbon source (Bringaud et al., 2015) and [U-^{13}C]-glycerol, respectively, were determined by ^{1}H-NMR spectroscopy.
Source data are provided as a Source Data file.

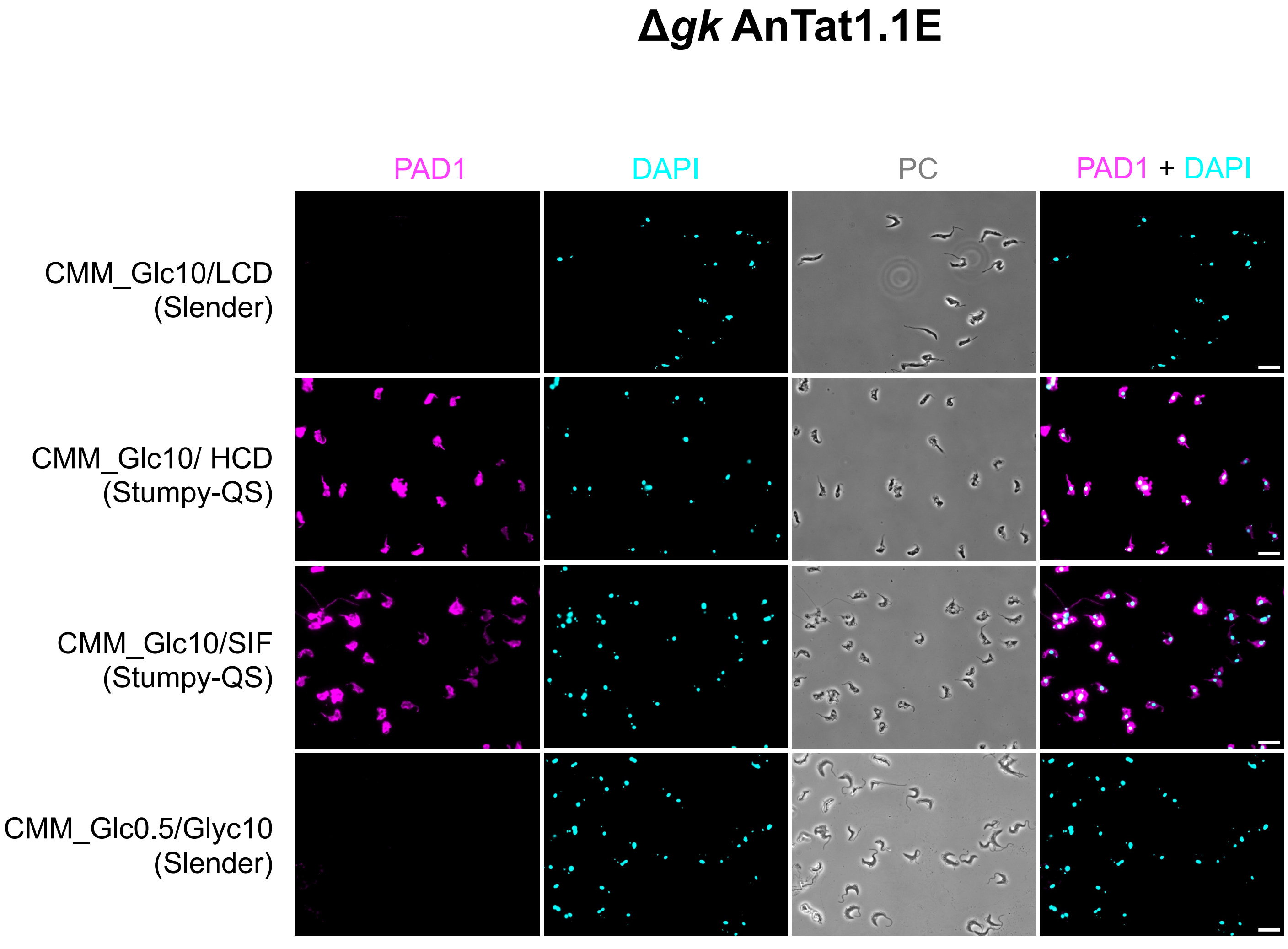

Supplementary figure 11. Glycerol kinase null mutant fails to differentiate to stumpy-Glyc when incubated with glycerol.
Immunofluorescence analysis of the glycerol kinase null mutant (Δ*gk* AnTat1.1E) incubated in (*i*) CMM_Glc10 and maintained as slender forms in CMM_Glc10 by daily dilution to prevent at high cell density, (*ii*) in CMM_Glc10 at high cell density without daily dilution (CMM_Glc10/HCD), (*iii*) in SIF-rich CMM_Glc10 spent medium derived from a stationary phase culture of AnTat1.1E-GFP::$PAD1_{UTR}$ (CMM_Glc10/SIF), and (*iv*) in low glucose (0.5 mM) CMM containing 10 mM glycerol (CMM_Glc0.5/Glyc10). PAD1 expression is revealed using anti-PAD1 (false colored magenta), nucleus and kinetoplast using DAPI (false colored cyan). PC Phase contrast. (n = 3 independent experiments). Scale bar = 20 µm.

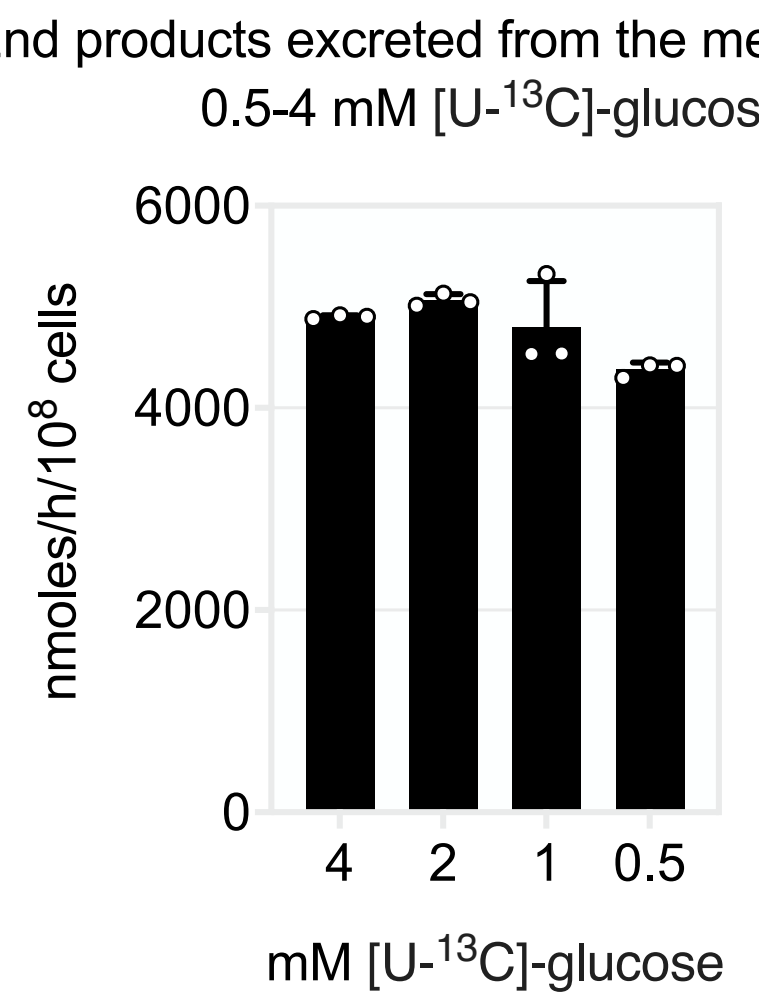

Supplementary figure 12. Analysis of glucose metabolism by ^{1}H-NMR analysis.
The parental AnTat1.1E cell line was incubated for 1 h in PBS/$NaHCO_3$ buffer containing 4, 2, 1 or 0.5 mM of uniformly ^{13}C-enriched glucose ([U-^{13}C]-glucose). The amounts of ^{13}C-enriched metabolic end products excreted in the spent medium from the metabolism of [U-^{13}C]-glucose were determined by ^{1}H-NMR spectroscopy.
Source data are provided as a Source Data file.

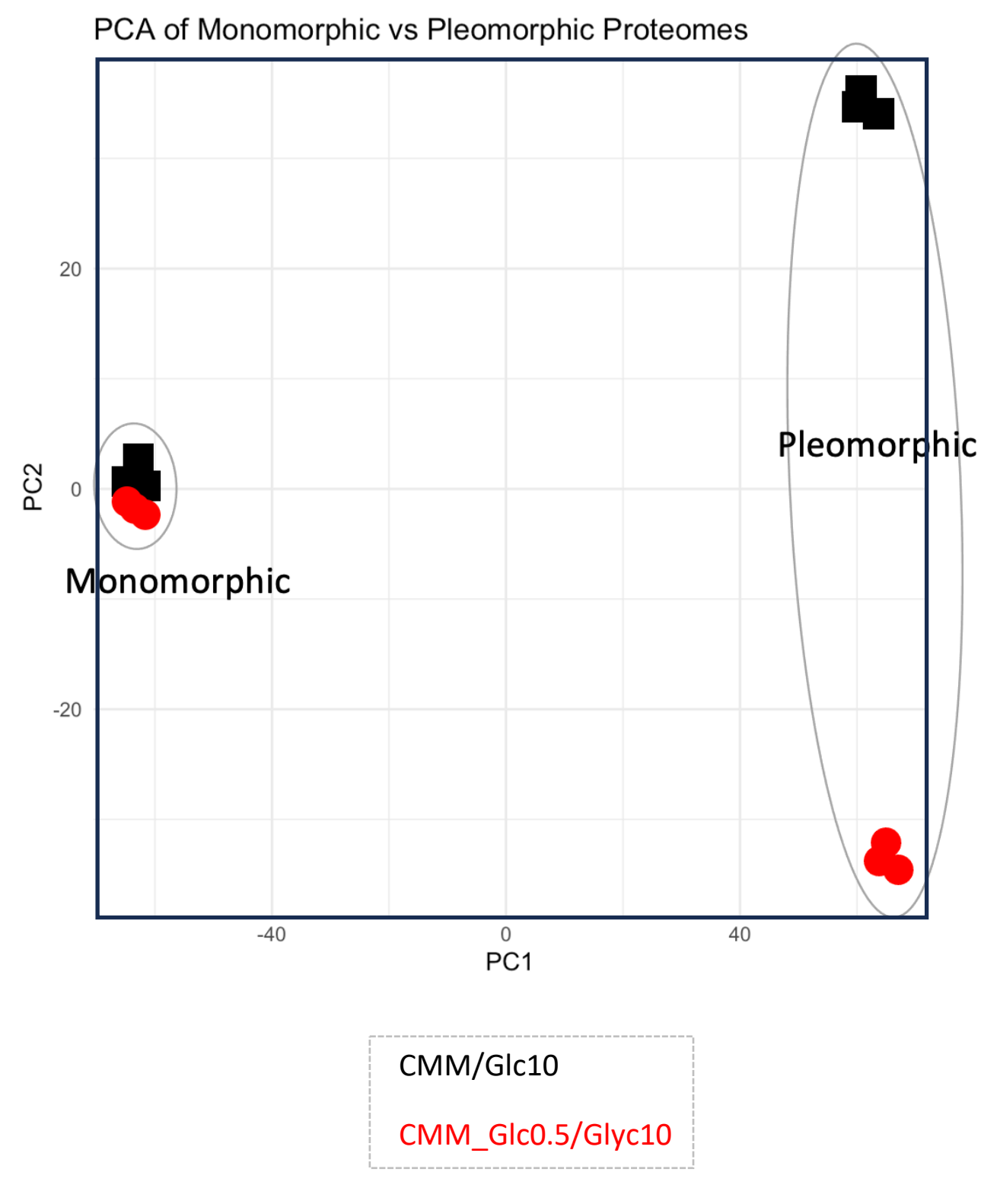

Supplementary figure 13. Principal Component Analysis (PCA) of proteomic profiles from monomorphic and pleomorphic strains.
The proteomic data generated from the Lister 427 monomorphic strain were previous described [1] and compared with those of the AnTat1.1 pleomorphic strain presented in Fig.1g. Both strains were grown under glucose-rich (CMM_Glc10) or glycerol-rich (CMM_Glc0.5/Glyc10) conditions. Each point represents a biological replicate.
Source data are provided as a Source Data file.

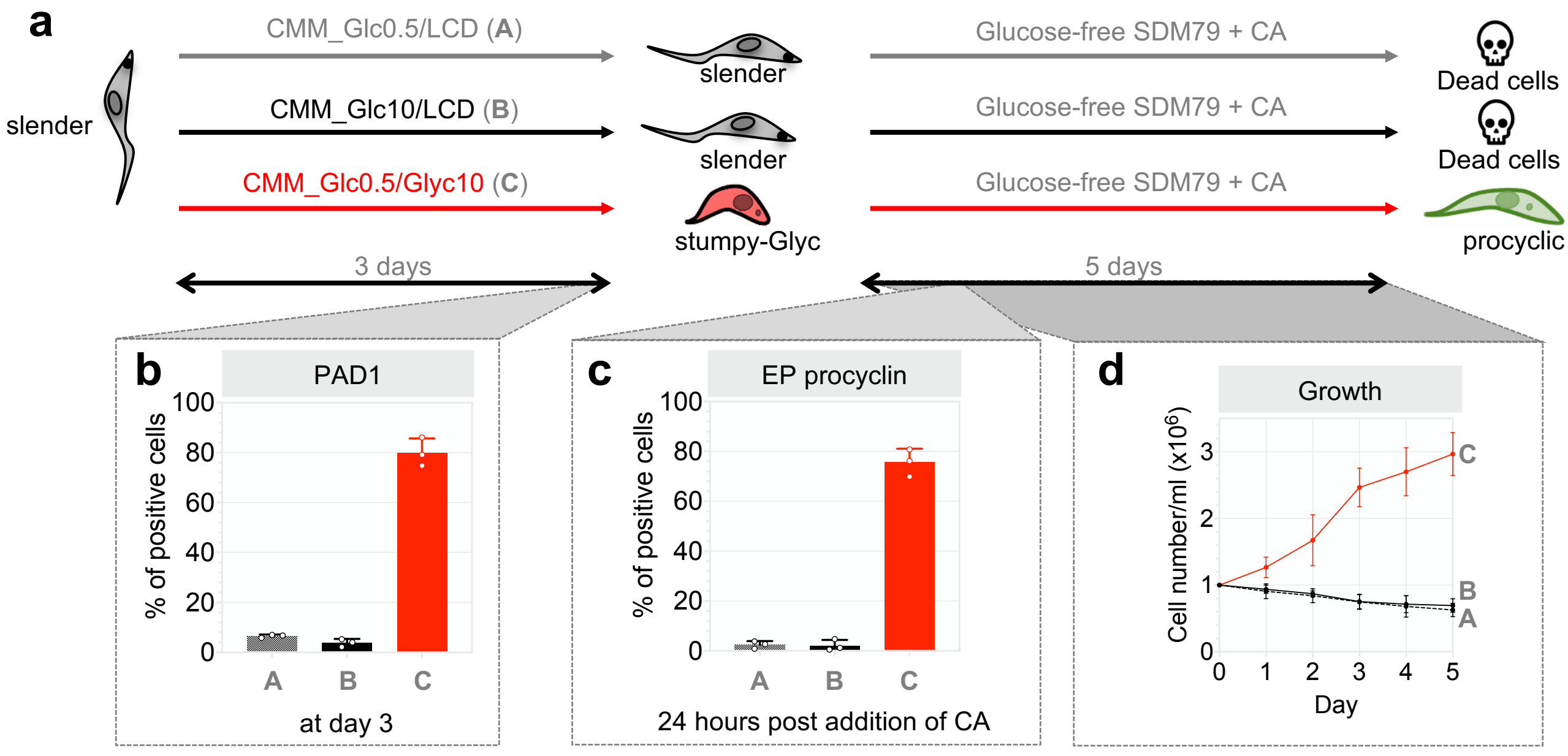

Supplementary figure 14. Low-glycerol levels do not alter slender-forms behavior.
a, Description of the experimental setup for the differentiation into PCF of slender forms of the parental AnTat1.1E-GFP::PAD1$_{UTR}$ cell line incubated in low-glucose (CMM_Glc0.5), glucose-rich (CMM_Glc10) and glycerol-rich (CMM_Glc0.5/Glyc10) conditions, and maintained at low cell density (LCD) by daily dilutions. The trypanosomes incubated for 5 days in these conditions were cultivated at 27°C in glucose-free SDM79 containing 6 mM CA. Data are presented in panels **b-d**, *i.e.* percentage of PAD1 positive (**b**) and EP positive (**c**) cells at day 0 and day 3, respectively, and cumulative growth in the PCF glucose-free SDM79 medium over five days (**d**). Each value corresponds to the mean (±SD) of 3 independent experiments (biological replicates).
Source data are provided as a Source Data file.

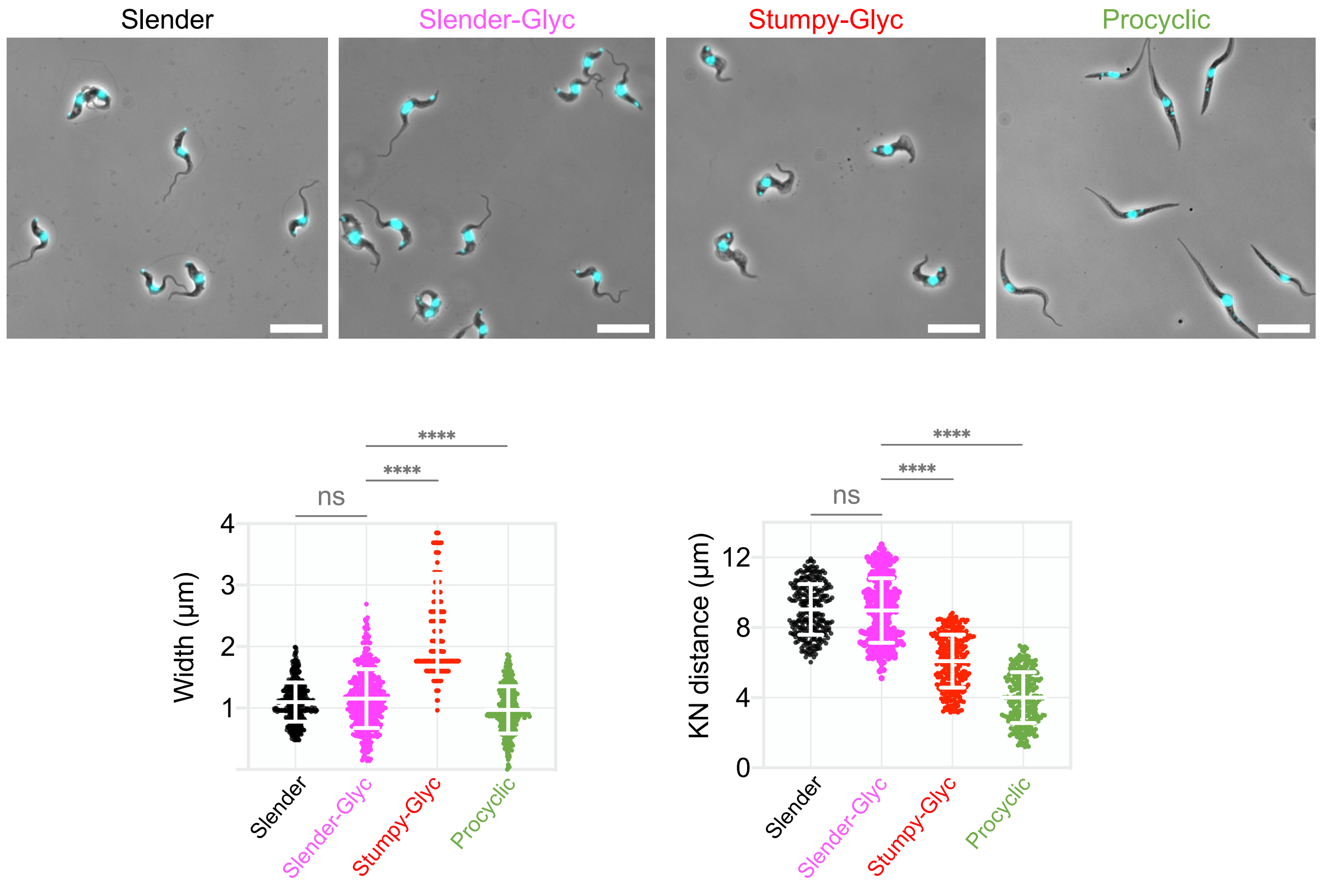

Supplementary figure 15. Slender-Glyc forms are slender-like forms.
Phase contrast microscopy images overlaid with DAPI fluorescence, highlighting the nucleus and kinetoplast (false-colored cyan) of distinct morphological forms: slender, slender-Glyc, stumpy-Glyc, and procyclic forms. Scale bar = 20 µm. Slender-Glyc morphology was confirmed by measuring the cell width and the distance between the kinetoplast and the nucleus (K-N) of 250-300 cells per group, using a previously described ImageJ macro (*n* = 3 independent experiments). Statistical significance was determined using two-sided Dunn's multiple comparison tests (****, $p < 0.0001$; ns, not significant).
Source data are provided as a Source Data file.

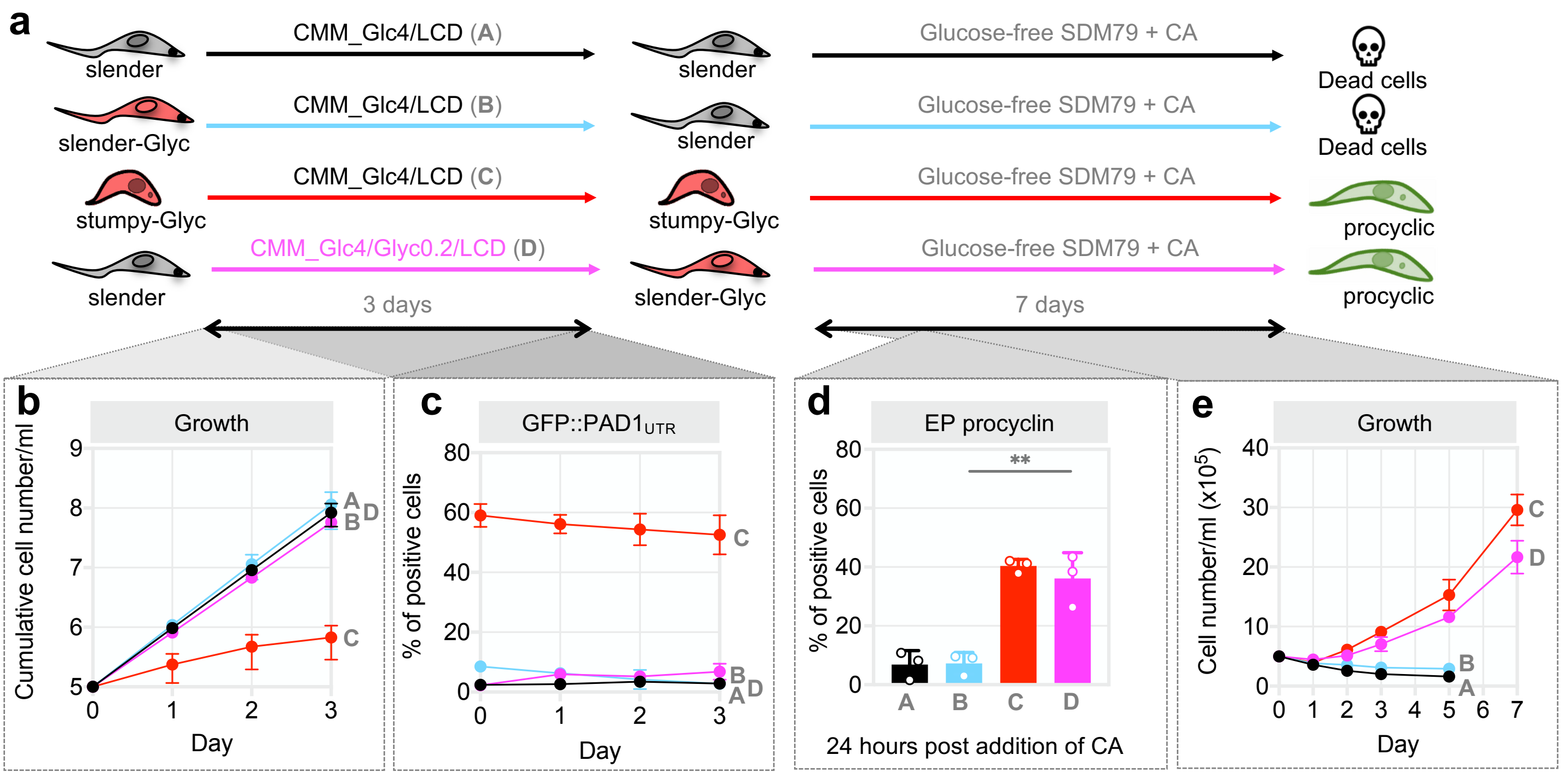

Supplementary figure 16. Slender-Glyc forms revert to slender in the absence of glycerol. **a**, Description of the experimental setup for the differentiation of different forms of the parental AnTat1.1E-GFP::PAD1$_{UTR}$ cell line into PCF. The goal is to determine whether slender-Glyc forms revert to slender forms when incubated in glycerol-free medium (B; CMM_Glc4), using subsequent differentiation to PCF as a readout. Slender forms incubated in CMM_Glc4 (A) serve as the negative control while stumpy-Glyc and slender forms incubated in CMM_Glc4 (C) and CMM_Glc4/Glyc0.2 (D), respectively, serve as positive controls. After 5 days in these conditions, trypanosomes were shifted to glucose-free SDM79 containing 6 mM CA and incubated at 27°C. Data are presented in panels **b-e**, *i.e.* continuous growth, with daily dilutions to keep cells at low density (**b**), expression of GFP::PAD1$_{UTR}$ (**c**) and EP procyclin (**d**), and cumulative growth in the PCF glucose-free SDM79 medium over seven days (**e**). Each value corresponds to the mean (±SD) of 3 independent experiments (n= 3 biological replicates). Statistical significance was determined using ordinary one-way ANOVA followed by Sidak's multiple comparisons test (**, p = 0.0011). Source data are provided as a Source Data file.

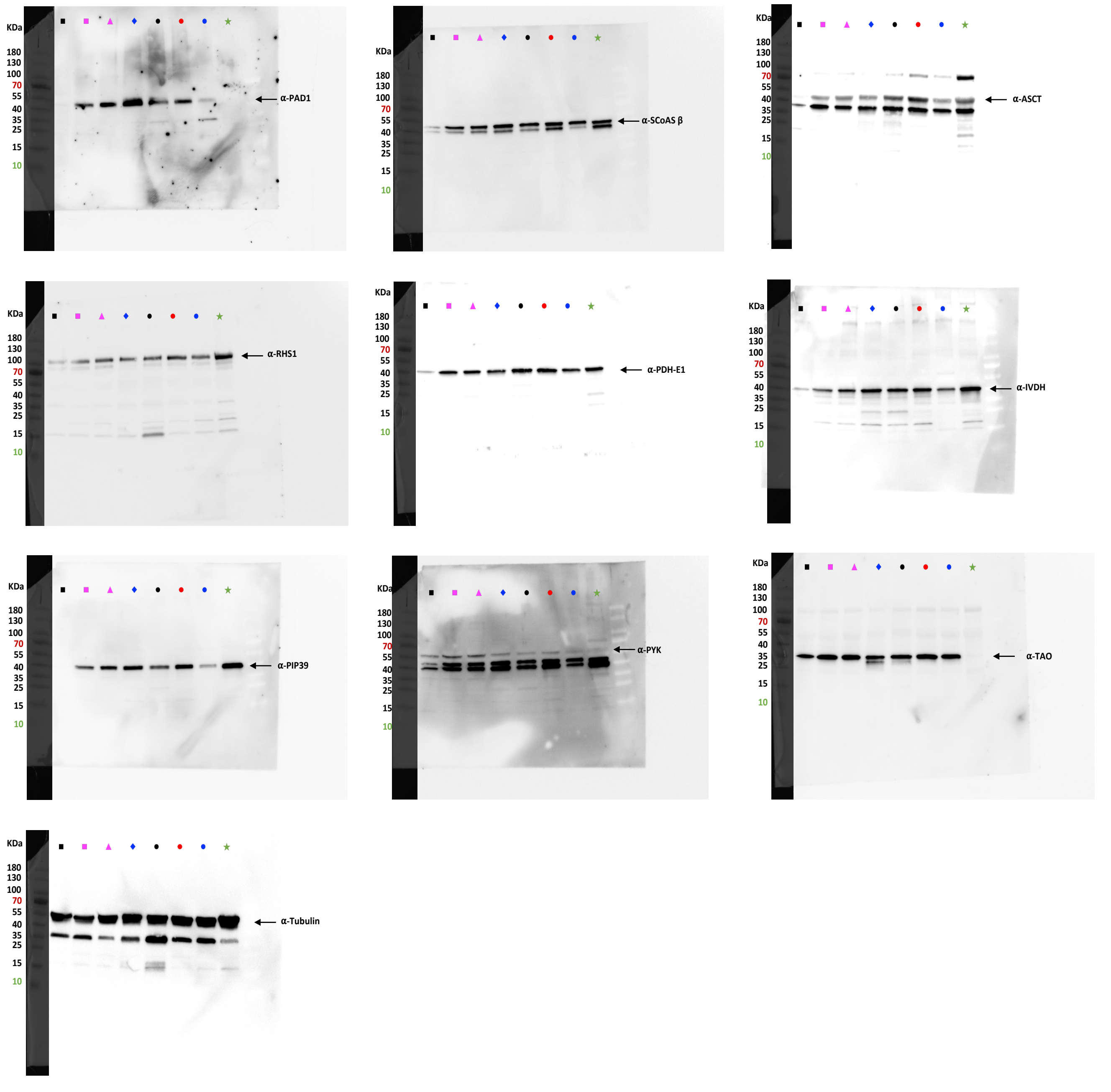

Full-length uncropped immunoblots corresponding to Supplementary Figure 8. Each panel shows a blot probed with a different antibody. The band corresponding to the target protein is indicated by an arrow.

Flow cytometry gating strategy

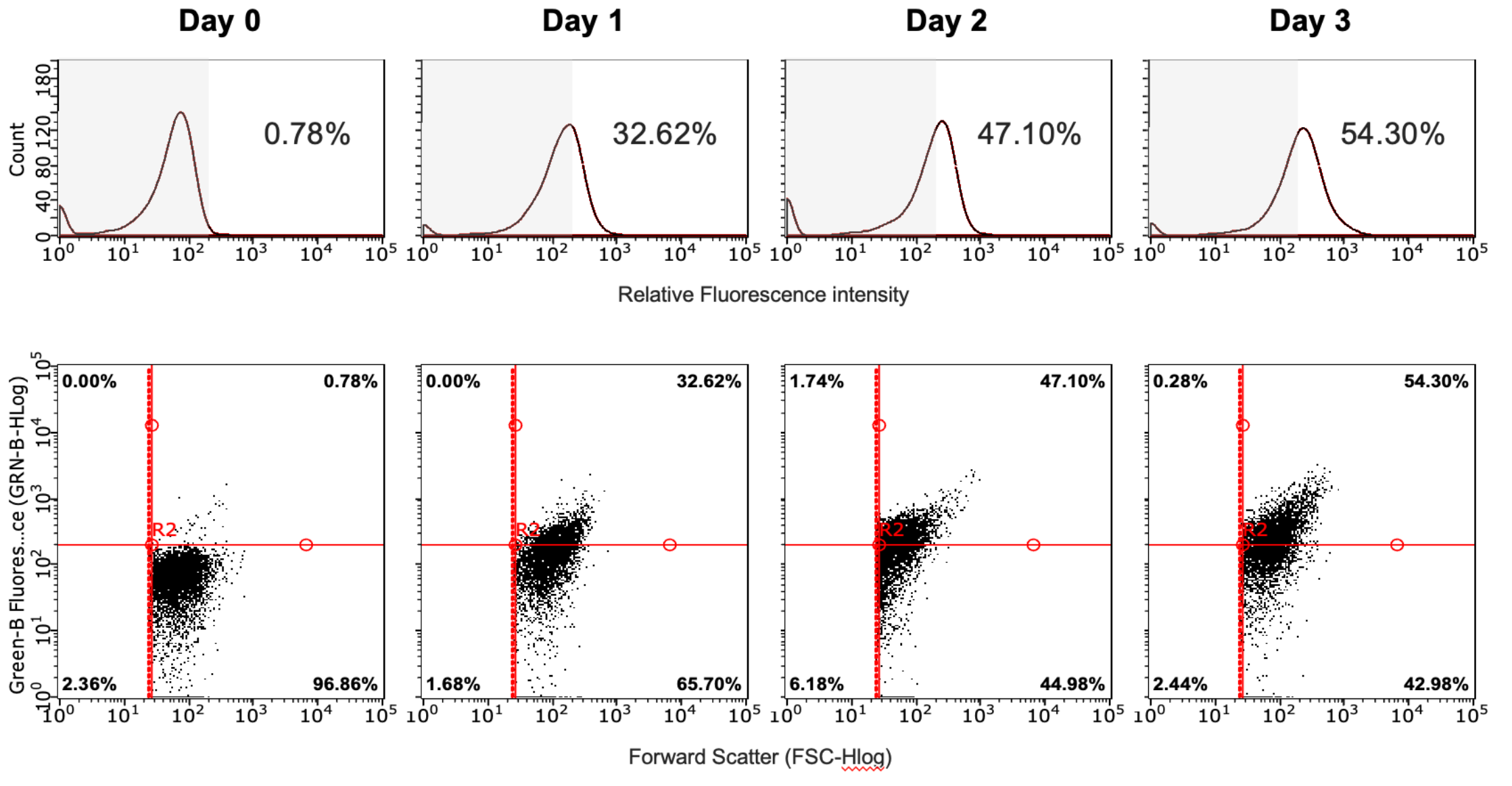

Representative flow cytometry gating strategy used to identify fluorescence-positive cells. Following exclusion of debris and low-quality events based on scatter characteristics, cells were gated according to fluorescence intensity. Positive gates were defined using the background fluorescence of negative control cells and were applied consistently across all samples. The same gating strategy was used for all fluorescence channels analyzed in this study, including green, red, and far-red fluorescence signals.
