# Supplementary material for: Glycerol metabolism triggers trypanosome differentiation into transmissible forms in mammalian tissue-like conditions
Source: Nat Commun. 2026 Jul 17;17:8766. doi: 10.1038/s41467-026-75670-w (PMC13493832; doi:10.1038/s41467-026-75670-w)
Supplement: Supplementary file 2 [file 41467_2026_75670_MOESM2_ESM.pdf]

Supplementary data 1: Proteomic analysis of the AnTat1.1E strain grown under different conditions.

Columns F, H, J, L, N, P and R show the abundance ratio between the conditions indicated in lane 1, with upregulated and downregulated proteins highlighted in red and blue, respectively. Columns G, I, K, M, O, Q and S show the corresponding *P* values, with statistically significant values highlighted in green. Columns U-AB show the mean abundance of the values used to calculate the abundance ratios.

Supplementary data 2: Proteins shared between different conditions.

These tables contain the data used to generate Supplementary Fig.5a-h. The presence or absence of each protein in the corresponding datasets are highlighted in green and red, respectively.
