# Supplementary material for: Glycerol metabolism triggers trypanosome differentiation into transmissible forms in mammalian tissue-like conditions
Source: Nat Commun. 2026 Jul 17;17:8766. doi: 10.1038/s41467-026-75670-w (PMC13493832; doi:10.1038/s41467-026-75670-w)
Supplement: Supplementary file 3 [file 41467_2026_75670_MOESM5_ESM.pdf]

nature portfolio

Corresponding author(s): Frédéric Bringaud

Last updated by author(s): 2026/06/22

Reporting Summary

Nature Portfolio wishes to improve the reproducibility of the work that we publish. This form provides structure for consistency and transparency in reporting. For further information on Nature Portfolio policies, see our Editorial Policies and the Editorial Policy Checklist.

Statistics

For all statistical analyses, confirm that the following items are present in the figure legend, table legend, main text, or Methods section.

n/a	Confirmed	
☐	☒	The exact sample size (*n*) for each experimental group/condition, given as a discrete number and unit of measurement
☐	☒	A statement on whether measurements were taken from distinct samples or whether the same sample was measured repeatedly
☐	☒	The statistical test(s) used AND whether they are one- or two-sided<br>*Only common tests should be described solely by name; describe more complex techniques in the Methods section.*
☒	☐	A description of all covariates tested
☒	☐	A description of any assumptions or corrections, such as tests of normality and adjustment for multiple comparisons
☐	☒	A full description of the statistical parameters including central tendency (e.g. means) or other basic estimates (e.g. regression coefficient) AND variation (e.g. standard deviation) or associated estimates of uncertainty (e.g. confidence intervals)
☐	☒	For null hypothesis testing, the test statistic (e.g. *F*, *t*, *r*) with confidence intervals, effect sizes, degrees of freedom and *P* value noted<br>*Give P values as exact values whenever suitable.*
☒	☐	For Bayesian analysis, information on the choice of priors and Markov chain Monte Carlo settings
☒	☐	For hierarchical and complex designs, identification of the appropriate level for tests and full reporting of outcomes
☐	☒	Estimates of effect sizes (e.g. Cohen's *d*, Pearson's *r*), indicating how they were calculated

Our web collection on statistics for biologists contains articles on many of the points above.

Software and code

Policy information about availability of computer code

Data collection	n/a
Data analysis	Most data were analyzed by Graphpad Prism version 11; Fiji version 2.14.0/1.54f; RStudio version 2025.05.1+513.

For manuscripts utilizing custom algorithms or software that are central to the research but not yet described in published literature, software must be made available to editors and reviewers. We strongly encourage code deposition in a community repository (e.g. GitHub). See the Nature Portfolio guidelines for submitting code & software for further information.

Data

Policy information about availability of data

All manuscripts must include a data availability statement. This statement should provide the following information, where applicable:

- Accession codes, unique identifiers, or web links for publicly available datasets
- A description of any restrictions on data availability
- For clinical datasets or third party data, please ensure that the statement adheres to our policy

The raw proteomic data generated in this study have been deposited in the PRIDE database via the ProteomeXchange Consortium under the identifier PXD056405 (https://www.ebi.ac.uk/pride/archive/projects/PXD056405). The processed proteomic data generated in this study are provided in the Supplementary data 1 and 2. Source Data are provided with this paper. Figures 1 is linked to Supplementary data 1. Supplementary figure 5 is linked to Supplementary data 2.

Research involving human participants, their data, or biological material

Policy information about studies with human participants or human data. See also policy information about sex, gender (identity/presentation), and sexual orientation and race, ethnicity and racism.

Reporting on sex and gender	n/a
Reporting on race, ethnicity, or other socially relevant groupings	n/a
Population characteristics	n/a
Recruitment	n/a
Ethics oversight	n/a

Note that full information on the approval of the study protocol must also be provided in the manuscript.

Field-specific reporting

Please select the one below that is the best fit for your research. If you are not sure, read the appropriate sections before making your selection.

☒ Life sciences ☐ Behavioural & social sciences ☐ Ecological, evolutionary & environmental sciences

For a reference copy of the document with all sections, see nature.com/documents/nr-reporting-summary-flat.pdf

Life sciences study design

All studies must disclose on these points even when the disclosure is negative.

Sample size	No formal sample size calculation was performed prior to the study. Sample sizes were instead determined based on commonly used standards in the field, as well as practical considerations including sample availability and experimental feasibility. The chosen sample sizes are consistent with those reported in previous studies employing similar experimental approaches, where comparable effect sizes and variability have been observed. In addition, multiple independent biological replicates were included to ensure reproducibility, and key findings were confirmed across independent experiments. The observed effect sizes were robust and statistically significant using appropriate statistical tests, described in the Materials & methods sections, suggesting that the sample sizes were sufficient to detect meaningful differences.
Data exclusions	No data were excluded from the analyses unless predefined quality control criteria were not met.
Replication	The number of biological replicates ranged from 3 to 9 depending on the experiment. Replicate numbers were adjusted according to data variability and the level of statistical robustness required.
Randomization	No randomization was required for the experiments performed in this study.
Blinding	Except for the dissection of tsetse batches, blinding was not relevant to the experimental design and was therefore not performed.

Reporting for specific materials, systems and methods

We require information from authors about some types of materials, experimental systems and methods used in many studies. Here, indicate whether each material, system or method listed is relevant to your study. If you are not sure if a list item applies to your research, read the appropriate section before selecting a response.

Materials & experimental systems

n/a	Involved in the study	
☐	☒	Antibodies
☐	☒	Eukaryotic cell lines
☒	☐	Palaeontology and archaeology
☐	☒	Animals and other organisms
☒	☐	Clinical data
☒	☐	Dual use research of concern
☒	☐	Plants

Methods

n/a	Involved in the study	
☒	☐	ChIP-seq
☐	☒	Flow cytometry
☒	☐	MRI-based neuroimaging

Antibodies

Antibodies used	All immune sera and monoclonal antibodies were generated by the research groups of the co-authors or, where indicated, were kindly provided by academic collaborators, and the dilutions used are indicated in brackets: the rabbit anti-GK (1:5,000, gift from P.A.M. Michels, Edinburgh, UK), the rabbit anti-PAD1 (an antipeptide antibody recognizing TbPAD1 amino acids CPKEPTRDAREAAPQ was generated in rabbits, by Covalab, as described before 16, and diluted 1:1,000), the rabbit anti-ASCT (1:100) 57, the rabbit anti-IVDH (1:100) 58, the mouse monoclonal anti-TAO (7D3, 1:100, gift from M. Chaudhuri, Nashville, TN, USA) 59, the mouse anti-tubulin (1:3,000, DM1A, Sigma T9026), the rabbit anti-SCoAS (1:500, gift from A. Zikova, Czech Republic) 60, the rabbit anti-PDH (1:500) 61, the rabbit anti-RHS1 (1:1,000) 62, the rabbit anti-PIP39 (1:1,000, gift from K. Matthews, Edinburgh, UK) 63, the rabbit anti-PYK (1:1,000, gift from P.A.M. Michels, Edinburgh, UK) 64, the mouse anti-PFR (1:5,000, gift from L. Kohl, Paris, France) 65. All the antibodies have been validated in the corresponding publication indicated.
Validation	All of these immune sera and monoclonal antibodies have been validated by western blotting and/or immunofluorescence analyses.

Eukaryotic cell lines

Policy information about cell lines and Sex and Gender in Research

Cell line source(s)	Trypanosoma brucei brucei, strain AnTat1.1E, from the WHO-CC biobank at ITM, Antwerp, Belgium (provided by J. Van Den Abbeele J, Antwerp, Belgium); AnTat1.1E-GFP::PAD1UTR cell line derived from the AnTat1.1E strain (provided by L. Figueiredo, Lisbon, Portugal); T. brucei Lister 427 90:13 strain, named here Lister 427, derived from antigenic type MiTat 1.2, clone 221a (provided by G. Cross, New York, USA); T. brucei EATRO1125.T7T strain (provided by E. Pays, Gosselies, Belgium); The 3T3F442a pre-adipocyte cells (Kerafast, USA).
Authentication	These cell line were not authenticated, except the 3T3F442a pre-adipocyte cells provided by a company (Kerafast, USA).
Mycoplasma contamination	No contamination
Commonly misidentified lines (See ICLAC register)	n/a

Animals and other research organisms

Policy information about studies involving animals; ARRIVE guidelines recommended for reporting animal research, and Sex and Gender in Research

Laboratory animals	Teneral (<72h) male Glossina morsitans morsitans tsetse flies (colony maintained at Institut Pasteur, Paris, France)
Wild animals	n/a
Reporting on sex	n/a as only males were used because infection of male teneral flies is regarded as the gold-standard experimental model (higher infection rates, reproducibility, and experimental control). In addition, comparing all the conditions with a sufficient analytic power in adult flies would have required a total number of flies and biological replicates that can't be supported by our tsetse colony.
Field-collected samples	n/a
Ethics oversight	Ethical approval was not required for flies.

Note that full information on the approval of the study protocol must also be provided in the manuscript.

Plants

Seed stocks	n/a
Novel plant genotypes	n/a
Authentication	n/a

Flow Cytometry

Plots

Confirm that:

☒ The axis labels state the marker and fluorochrome used (e.g. CD4-FITC).

☒ The axis scales are clearly visible. Include numbers along axes only for bottom left plot of group (a 'group' is an analysis of identical markers).

☒ All plots are contour plots with outliers or pseudocolor plots.

☒ A numerical value for number of cells or percentage (with statistics) is provided.

Methodology

Sample preparation	2-5 x 106 cells were washed twice in PBS prior to fixing in 500 µl 4% paraformaldehyde in PBS for 20 min at RT. Cells were then washed 3x in PBS and resuspended in 4% BSA:PBS for 30 min. Cells were then resuspended in primary antibody diluted in 4% BSA:PBS, anti-EP procyclin (Cedar Lane laboratories, diluted 1:500) or anti-PAD1 (diluted 1:500), and were incubated overnight at 4oC. The cells were washed twice in PBS and were resuspended in secondary antibody diluted in 4% BSA:PBS (anti-mouse Alexa Flour 647&488 were diluted 1:500). Cells were washed twice in PBS and were resuspended in 500 µl PBS.
Instrument	Guava EasyCyte Flow Cytometer (Merck Millipore)
Software	Guava® InCyte
Cell population abundance	Analyzed cell populations were within 1000s to 100000s events.
Gating strategy	Following exclusion of debris and low-quality events based on scatter characteristics, cells were gated according to fluorescence intensity. Positive gates were defined using the background fluorescence of negative control cells and were applied consistently across all samples. The same gating strategy was used for all fluorescence channels analyzed in this study, including green, red, and far-red fluorescence signals.

☒ Tick this box to confirm that a figure exemplifying the gating strategy is provided in the Supplementary Information.
