# Supplementary material for: Glycerol metabolism triggers trypanosome differentiation into transmissible forms in mammalian tissue-like conditions
Source: Nat Commun. 2026 Jul 17;17:8766. doi: 10.1038/s41467-026-75670-w (PMC13493832; doi:10.1038/s41467-026-75670-w)
Supplement: Supplementary file 4 [file 41467_2026_75670_MOESM6_ESM.pdf]

nature portfolio

Peer Review File

Glycerol metabolism triggers trypanosome differentiation into transmissible forms in mammalian tissue-like conditions

Corresponding Author: Dr Frederic Bringaud

This file contains all reviewer reports in order by version, followed by all author rebuttals in order by version.

Version 0:

Reviewer comments:

Reviewer #1

(Remarks to the Author)
The paper includes experiments that document the effects of varying glycerol concentrations on the capacity of African trypanosomes to generate potentially transmissible forms in vitro. The argument is made that interstitial glycerol in the skin and adipose tissue environments is sufficient to stimulate trypanosomes to develop to an 'intermediate-gly' form that is proliferative yet able to infect tsetse. This is proposed to represent a dominant contributor to the ability of trypanosomes to transmit, particularly when at low parasitemia and when the already described quorum sensing process may be less likely to operate (as might occur in chronic infections in livestock) - the so-called 'transmission paradox'. The experiments are well carried out and thorough and may provide a novel insight into transmission biology of the parasite. However, there remain important questions over the physiological significance of the results when in an in vivo setting, so that the claimed dominant role for this mechanism in transmission is currently insufficiently supported.

General and specific points are detailed below.

1. Glycerol concentrations and physiological relevance. In vivo concentrations of glycerol in adipose tissue are reported to be 0.16 - 0.18 mmol (Vestergaard, et al 2013; the higher levels reported by Maggs et al have been shown to be inaccurate). Information on skin glycerol levels are not clear, but glucose4/glyc 0.25 (proposed as 'skin like' in the manuscript) could be reasonable in subcutaneous adipose. However, this might be beyond the normal reach of tsetse feeding (reported to be 1-2mm; Reuter et al. Nat communications 2023). Are values for glycerol and glucose available for skin specifically (i.e. in the dermis/epidermis)?

More importantly, these cited levels of physiological glycerol are more than 50 fold lower than the 10mM glycerol used for all of the data in Figures 1-4, and at the lower end of the concentrations used in Figure 6. The authors clearly acknowledge that artificially high concentrations of glycerol are used in Figures 1-4, where there is in vitro development to 'stumpy-gly' forms supported by high quality experiments. However, given the unusually high glycerol concentrations, the biological relevance for much of the presented results are unclear (several other unphysiological metabolic assaults could also promote stumpy-like formation, perhaps?). For example, the significance of the proposed lifespan extension of glycerol generated stumpy forms and their delayed commitment (Figure 2) is not clear since these would not arise at physiological glycerol levels according to the authors' model. The interpretation of this data on commitment/reversibility should also be more cautious, since the experiments cannot distinguish the regrowth of uncommitted individual cells from outgrowth of a small founder population of cells that never arrested. Also, would SIF stumpy cells persist longer if exposed to high or physiological glycerol media (i.e. is it the environment or the cell origin that dictates an extended lifespan?). Did I miss this data?

2. Where closer to physiological concentrations of glycerol (0.1-0.5 mmol) are used to generate 'intermediate-gly' forms, procyclic forms arise in vitro in response to cis aconitate (albeit quite inefficiently and, at 0.1mmol, without subsequent outgrowth as viable procyclic forms)(Figure 6). Again, these differentiated procyclic forms may arise from proliferative 'intermediate-gly' cells as proposed or a small subset of non-proliferative cells in the population and so more caution is needed in the interpretation. Nonetheless, a limited level of procyclic development is possible at low concentrations of glycerol in vitro (it is a shame that 0.2mmol glycerol is not tested as this appears most representative of adipose tissue levels). For the tsetse infection studies, directly comparable glycerol concentrations were (frustratingly) not used between the in vitro and tsetse experiments (and a range of concentrations was not tested in flies which would assist comparison with the in vitro data). Nonetheless, with 0.25 mmol glycerol-generated 'intermediate-gly' parasites, some tsetse infectivity was

observed (Figure 6h) - although the ‘equivalence’ of intermediate-gly and SIF induced stumpy forms with respect to infectivity may be overstated (9.7% vs 17.3% is stated as being equivalent perhaps due to the variability in the data).

3. Importantly, however, teneral flies are used for these fly studies which these authors have previously shown (Ngoune et al, eLife 2023) are rather permissive for parasite establishment. The experiment should therefore be extended to comparing the infection of adult flies by (i) intermediate-gly forms, (ii) proliferating slender forms and (iii) stumpy forms generated by quorum sensing. Maturation to salivary gland metacyclic forms would also be informative. Combined, this would provide evidence of the physiological significance of intermediate-gly parasites in infections likely to be relevant in the field.

4. The studies are fully restricted (with the exception of tsetse infectivity) to in vitro analysis. With the availability and elegant validation of mutants (GK, AQP1-3 null mutants) that render any glycerol response inactive (Figure 3) there is the opportunity to explore the relative contribution of glycerol sensing and quorum sensing driven differentiation to the infection in vivo. This would provide important physiological insight into the glycerol effect beyond in vitro studies, rather than being beyond the scope of the current study. This is relevant because stumpy forms are reported to be prevalent in adipose tissue and this could address whether these arise from quorum sensing or are glycerol induced, or a combination of the two. The relative contribution of the respective mechanisms (quorum sensing, glycerol) is important to address if glycerol stimulation is proposed to resolve the transmission paradox as claimed, and the model presented in Figure 7 should address early as well as chronic infection (a switch from quorum sensing stumpy generation to glycerol stimulated intermediate-gly forms, or a combination of the two mechanisms in chronic infection?)

5. Minor point. In Figure 5 why is panel a expressed as a unit value and panel b as a percentage value? There is reference made to a red circle indicating hydrogen atoms that I cant see . The legend describes the adipocyte production of glycerol as 1mM while the figure says up to 1mM, and the text says around 1mM. Could you provide the range observed?

Overall, there is an impressive amount of high-quality data presented and a hypothesis is provided to augment existing models to explain transmission of trypanosome parasites at low parasitemia. This could combine with the established quorum sensing mechanism to explain how transmission can be sustained in chronic infections. Other mechanisms are possible however as an alternative or in addition (e.g. peptides generated by inflammatory proteases from macrophages and neutrophils could generate stumpy forms at low parasitemia, as could local concentrations of parasites trapped in the tissues). As things stand, while I am impressed with the data presented, the relevance of glycerol in infections and for transmission need much further experimental justification to support its claimed dominant role in parasite development in vivo.

Reviewer #2

(Remarks to the Author)
Review of the manuscript: “Glycerol metabolism triggers trypanosome differentiation into transmissible forms in mammalian tissue-like conditions”
The submitted manuscript presents an interesting and novel observation: the presence of glycerol in the culture medium enables bloodstream-form (BF) Trypanosoma brucei to differentiate into stumpy-like cells, even at lower cell densities than typically required for the canonical stumpy induction factor (SIF)-dependent pathway.
The authors clearly recognize the significance of their findings and have implemented a comprehensive series of controls that confirm the role of glycerol in triggering differentiation into stumpy-like forms. Their hypothesis—that the presence of glycerol promotes the differentiation of long slender (LS) BF into what they term “stumpy-glyc” cells—is supported by carefully designed experiments involving precise cell counts and detection of the PAD1 marker (via IFA). These stumpy-glyc cells are shown to differentiate into procyclic forms in vitro and are also capable of infecting tsetse flies. The identification of these forms is based primarily on PAD1 expression, changes in cell morphology (size and nucleus-kinetoplast distance), and cell cycle arrest in G1/G0 phase.
Using a wide range of culture conditions, including varying concentrations of glucose and glycerol, the authors demonstrate that stumpy-glyc forms may have physiological relevance. They propose that this population could help explain the so-called “transmission paradox”, the ability of trypanosomes to be transmitted between hosts even at low parasitemia levels, potentially due to these forms residing in tissues like the skin, where glycerol concentrations are higher.
They also offer an elegant reinterpretation of prior observations published by Schuster et al., where LS BF cells were found to differentiate into procyclic forms in glycerol-containing media.
The authors further conducted proteomic analyses, comparing expression profiles across different cell forms. These data demonstrate that the stumpy-glyc proteome is clearly remodeled and partially overlaps with that of SIF-induced stumpy (stumpy-QS) forms. However, these proteomic results do not yet elucidate the molecular mechanism by which glycerol promotes differentiation, and the signaling or metabolic trigger remains unknown.
While reading the manuscript, I repeatedly considered whether the observed effects might be due not to the presence of glycerol per se, but rather to a reduction in glucose availability or its metabolism. Many experiments are conducted in conditions with 0.5 mM glucose and 10 mM glycerol, which may significantly limit glycolytic flux. Even in later experiments using more physiologically relevant glucose levels (4 mM), it remains possible that differentiation is triggered by reduced glucose metabolism rather than glycerol itself.
I am not fully convinced that glycerol is the primary driver of differentiation. It may instead serve as a supplemental carbon and energy source, helping to sustain viability under conditions of glucose limitation. Reduced glycolytic activity may influence the ADP/ATP and NAD/NADH ratios, which are known to affect metabolic signaling. The authors themselves have previously reported increased succinate and acetate secretion in the presence of glycerol, consistent with altered

metabolism—potentially to maintain redox balance (NADH/NAD) in the glycosome and/or enhance ATP production through the acetate shuttle in mitochondria.
The discussion would benefit from further elaboration on possible mechanisms through which glycerol may influence metabolism and gene expression to produce the observed stumpy-glyc phenotype. For example, prior studies have identified a glycerol-responsive element in the 3′UTR of certain trypanosome transcripts; although these findings are older, they might be worth mentioning as a potential regulatory mechanism or not.
That said, these comments do not detract from the quality of the work or the significance of the findings. Rather, they highlight the importance and open-ended nature of the questions raised regarding both the physiological relevance and underlying molecular mechanisms.

Specific comments on figures and data presentation:
Figure 1
The growth arrest of long slender (LS) BF cells in CMM_Glc0.5/Glyc10 mM is reminiscent of the findings by Pineda et al., where monomorphic LS cells required adaptation to glycerol-containing media before resuming growth. Although a different (pleiomorphic) strain is used here and the earlier studies involved glucose uptake inhibitors, it may be worth considering whether a similar adaptation mechanism is at play—i.e., arrest due to a sudden drop in glucose, followed by adaptation to glycerol utilization. Is it possible to compare proteomes (if available) of monomorphic and pleiomorphic strains grown under CMM_Glc0.5/Glyc10 mM?
Based on PCA plots, stumpy-QS and stumpy-glyc forms appear closely related. In addition to the Venn diagrams provided, it might be useful to include a comparative analysis of protein-level changes—identifying shared versus unique changes—and a correlation coefficient to quantify the similarity between these two conditions.
Figure 2
The manuscript notes that cell death was assessed visually. If a live/dead assay was performed, including this data would strengthen the conclusion that cells remain viable under Glc0.5/Glyc10 mM conditions.
• One skull icon appears misplaced in the top graph—cells at 10^6 died on day 3.
• In panel 2d, the authors might consider discussing these results in light of Larcombe et al. (2023, PNAS), which suggested that irreversible commitment to differentiation occurs before PAD1 expression.
Figure 3
Verification of successful gene replacements for the GK and AQP knockout cell lines by PCR should be shown in the Supplementary Materials. This is particularly relevant given Pineda et al.'s findings, where RNAi against GK led to undetectable protein levels by Western blot, yet residual GK activity was suggested by the phenotype.
Given the essential roles of AQP and GK in glycerol metabolism, it is striking that the knockout lines are viable and proliferative in Glc0.5/Glyc10 conditions. This implies that 0.5 mM glucose may be sufficient to support cell growth of LS BF cells. Including a control with Glc0.5 alone would be helpful. What happens when wt and mutant cells are cultured in 0.5 mM glucose without glycerol? Do they arrest or die?
In Figure 3D, were the knockout cells split daily? If not, what happens when they reach densities above 1×10^6 cells/mL? Do they arrest (perhaps due to activation of the SIF pathway) or die (due to glucose exhaustion)?
Interestingly, the GK and AQP knockout cells arrest under CMM_Glc10/SIF at higher densities and later timepoints than WT or rescued lines. Do the authors have any explanation for this delayed response?
I am also unclear on the rationale for using smLCD medium. Without metabolomic analysis, it is difficult to interpret the glucose and glycerol content of this medium. Unless there is a specific justification, the added value of using smLCD is uncertain. If its inclusion is important, a clearer explanation would be appreciated.
Figure 4
The legend and main text should clarify how GK knockout cells were handled. In CMM_Glc0.5/Glyc10 mM, the cells remain proliferative—were they diluted daily for the first three days? At what density was differentiation to PCFs induced? Were they allowed to reach high densities (e.g., 1×10^6 cells/mL)?
Figure 6
What would happen under conditions with both low glucose and low glycerol (e.g., CMM_Glc0.5/Glyc0.5)? Figure 3 shows that GK mutant cells are viable with 0.5 mM glucose and 10 mM glycerol, suggesting that minimal glucose is sufficient to maintain viability. Would the cells arrest or be capable of differentiating into PCFs under more restricted carbon source availability?
Figure 6H is described as showing that CMM_Glc4/Glyc0.25-derived stumpy cells and those generated under CMM_Glc4 + SIF conditions establish infections in the tsetse midgut at comparable levels (line 352). However, no statistical analysis is provided to support this statement. At first glance, the CMM_Glc4/Glyc0.25-derived stumpy cells appear to have a reduced capacity to establish infections compared to the SIF-induced stumpy cells.
I recommend that the authors re-evaluate this conclusion by performing appropriate statistical analysis to assess whether the observed difference is significant. If the difference is indeed not statistically significant, this should be clearly stated. Otherwise, the claim of "comparable infection levels" should be revised accordingly.

Final remarks:
Overall, the manuscript presents an intriguing and important observation regarding the potential role of glycerol (and/or glucose limitation) in inducing trypanosome differentiation. However, the precise mechanism—whether metabolic, signaling-based, or both—remains unclear. A more thorough discussion of alternative interpretations, particularly those involving glucose metabolism and nutritional stress, would significantly strengthen the manuscript and its impact.

Reviewer #3

(Remarks to the Author)
I co-reviewed this manuscript with one of the reviewers who provided the listed reports. This is part of the Nature Communications initiative to facilitate training in peer review and to provide appropriate recognition for Early Career Researchers who co-review manuscripts.

Version 1:

Reviewer comments:

Reviewer #1

(Remarks to the Author)
This is an improved manuscript that addresses some but not all of the previous queries. In particular, valuable additional data is presented that indicates that stumpy forms generated by SIF show an extended lifespan with low glycerol concentrations, which likely has relevance in vivo. Similarly, valuable further glycerol concentrations have been evaluated with respect to tsetse infectivity that provide better correspondence with the in vitro data on differentiation.

However, there remain important cautions that we feel must be more explicitly addressed to support the claims in the paper and to avoid confusion in the literature. We detail these below

1. The description and characterization of stumpy-gly forms. As stated before, these experiments are all high quality and convincing. But nonetheless the analyses all focus on a cell form that is an experimental artefact (because glycerol levels are used that are far higher than exists in vivo). Whilst acknowledged, this needs to be absolutely explicit because otherwise I fear there could be misinterpretation by readers who are less careful than the authors of this manuscript. We suggest that throughout this section (figures 1-4) and relevant text and also subheadings, the authors consistently add the word 'supraphysiological' before 'glycerol' and emphasize the unphysiological nature of the experiments. Otherwise, the more meaningful experiments carried out with lower (possibly physiological) glycerol concentrations may lose impact and readers may incorrectly conclude 'glycerol can drive stumpy formation', which is physiologically incorrect.
2. The relevance of the low glycerol concentrations in vivo. The paper often refers to adipose levels of glycerol, but – as acknowledged in the rebuttal- the levels of glycerol in the skin are unknown. Likewise, levels could be significantly affected by the depth of feeding by the tsetse (as previously discussed in our original review). Here, the manuscript is too loose concerning whether they are addressing the 'transmission paradox' with respect to human or livestock infection. In humans the skin and subcutaneous fat is within reach of the tsetse proboscis, but in livestock (which is where the proposed transmission paradox is most important as this is the major focus of parasite infections) the dermal layer can extend 3.4mm-over 5mm, well beyond the tsetse feeding depth. The manuscript needs to be explicit about whether human or livestock transmission is being addressed in their arguments (and for humans a different parasite- Trypanosoma brucei gambiense- is the main disease agent, not T. b. brucei used here). They should also discuss the depth of the dermis of cattle in relation to their model.
3. In relation to point 2, it is stated trypanosome infections could elevate glycerol levels through adipolysis. But the evidence for this is from high parasitemia mouse infections (Machado et al, 2023) in white adipose tissue, which the authors state is not field relevant (not being in skin and in high parasitemia rodent infection). Self-evidently, the low parasitemias that invoke the transmission paradox are difficult to explain by trypanosome-generated glycerol levels that have only been observed at high parasitemia. Adipolysis by local concentrations of parasites could contribute of course, but then QS can also operate, and there is no paradox. Consequently, trypanosome driven adoplysis as a mechanism for elevated glycerol generation cannot readily provide a solution to the proposed transmission paradox.
4. The response to lower concentrations of glycerol appears exquisitely sensitive. This is evident particularly in Figure 6f, where 0.1mM and 0.2mM glycerol generate very important differences in response (compare condition E which does not grow with condition F that does). This sensitivity matters because it is in this concentration range where normal physiological levels of tissue glycerol are expected according to the literature (0.16mM -0.18mM).
5. Tsetse infection experiments. We found the arguments for using teneral flies weak and rather undermined the main proposition of the paper (i.e. that the glycerol generated slender forms explain the transmission paradox). Most labs use teneral flies for trypanosome infections precisely because they are unnaturally permissive and so maximise the chances of progressing and studying the life cycle in flies. But the claim in this manuscript is that the transmission paradox can be resolved by glycerol stimulated parasites, which requires the infections to be most relevant to the field (not "they can do it" but "do they do it in the real world"?). This is surprising because the authors themselves address exactly this point in their earlier paper focused on the infectivity of slender and stumpy trypanosomes, where they show slender cells infect adult flies exceptionally poorly and discuss that teneral flies are not important in field transmission ("the impact of teneral flies on trypanosome transmission may be limited, if not incidental; "Ngoune et al, 2025 PMID: 40522086). Not doing this experiment actively weakens the conclusions of the paper.

Overall, the experiments remain of good quality (and have been improved with the revision) but there are significant question marks over the biological meaning and interpretation of them, and there is a failure to address some issues directly. The presented model is valuable, but as things stand it is not as compelling as claimed. Without wishing to impose the burden of yet more experimental effort, the authors need to be far more cautious in their interpretations and more explicit concerning where there is a lack, or limitation, of knowledge. This would avoid possible misinterpretation of the interesting data.

Minor point

line 566, ref 16, should be ref 17.

Reviewer #2

(Remarks to the Author)
The resubmitted manuscript adequately addresses the reviewers’ comments and incorporates several important additions and revisions. In particular, my previous concern regarding the possibility that glucose limitation itself could account for the observed phenotype was addressed through newly included control experiments. These additional data demonstrate that low glucose conditions alone are not sufficient to induce differentiation and that glycerol metabolism is the key trigger underlying the observed effect.
The authors also strengthened the physiological relevance of the study by performing new experiments under conditions that better mimic the composition of dermal interstitial fluids (~0.2 mM glycerol and 4 mM glucose). Under these tissue-like conditions, they describe a novel proliferative transmissible form of T. brucei, termed “slender-Glyc,” which appears distinct from classical stumpy forms while remaining competent for differentiation into procyclic forms and transmission to tsetse flies. Additionally, the revised manuscript includes additional experiments excluding the possibility that the observed transmission competence is due to rare contaminating stumpy cells. The manuscript further benefits from several new datasets and controls, including validation of mutant cell lines.

Overall, the revised manuscript is strengthened, and I have no further concerns. I would like to congratulate the authors on a meticulous and experimentally robust study that opens new avenues for defining the molecular mechanism underlying glycerol-induced differentiation, identifying molecular markers of the newly defined form, confirming its existence in vivo, and helping to explain the long-standing transmission paradox.

Reviewer #3

(Remarks to the Author)
I co-reviewed this manuscript with one of the reviewers who provided the listed reports. This is part of the Nature Communications initiative to facilitate training in peer review and to provide appropriate recognition for Early Career Researchers who co-review manuscripts.

Open Access This Peer Review File is licensed under a Creative Commons Attribution 4.0 International License, which permits use, sharing, adaptation, distribution and reproduction in any medium or format, as long as you give appropriate credit to the original author(s) and the source, provide a link to the Creative Commons license, and indicate if changes were made.
In cases where reviewers are anonymous, credit should be given to 'Anonymous Referee' and the source.
The images or other third party material in this Peer Review File are included in the article’s Creative Commons license, unless indicated otherwise in a credit line to the material. If material is not included in the article’s Creative Commons license and your intended use is not permitted by statutory regulation or exceeds the permitted use, you will need to obtain permission directly from the copyright holder.
To view a copy of this license, visit https://creativecommons.org/licenses/by/4.0/
